# Synthesis and Characterization of Sulfonamide‐Schiff Bases, and Investigation of Cytotoxic, Antioxidant, HDAC, and Apoptotic Activities in Human Colon Cancer Cells (DLD‐1 and HT‐29)

**DOI:** 10.1002/ardp.70235

**Published:** 2026-04-10

**Authors:** Seda Mesci, Berna Kocaman, Aliye Gediz Erturk, Emine Bagdatli, Burak Yazgan, Tuba Yildirim

**Affiliations:** ^1^ Project Coordination and Guidance Office, Rectorate Hitit University Çorum Turkey; ^2^ Department of Biotechnology, Institute of Science Amasya University Amasya Turkey; ^3^ Department of Chemistry, Faculty of Science and Arts Ordu University Ordu Turkey; ^4^ Department of Medical Services and Techniques, Sabuncuoğlu Serefeddin Health Services Vocational School Amasya University Amasya Turkey; ^5^ Department of Biology, Faculty of Art and Science Amasya University Amasya Turkey

**Keywords:** antioxidant, apoptotic, colon cancer, cytotoxic, HDAC, Schiff base, sulfonamide

## Abstract

Comprehending the intricate mechanisms of apoptosis and its interaction with cytotoxic, antioxidant, and HDAC activities is imperative for devising effective cancer therapies. Sulfonamides and Schiff bases are compounds of pharmacological importance with known anticancer activity. Our study aimed to investigate the cytotoxic, antioxidant, HDAC, and apoptotic activities of new sulfonamide‐Schiff bases in human colon cancer cells (DLD‐1 and HT‐29). New sulfonamide‐derived Schiff base compounds (**3a**–**d**) were synthesized from the condensation of sulfamethoxypyridazine (**1**) and various aromatic aldehydes, and were characterized by FTIR, NMR (^1^H and ^13^C/APT), UV‐Vis., and mass spectroscopy. Sulfonamide‐derived Schiff bases **3a–d** and compound **1** exhibited significant anticancer activity against colorectal cancer cell lines (DLD‐1, HT‐29). In the MTT assay, **3c** was most active in DLD‐1 (viability: 37.7%, IC₅₀ = 3.94 µM) and **3b** in HT‐29 (viability: 46.6%, IC₅₀ = 3.26 µM). In the WST‐8 assay, **3c** was strongest in DLD‐1 (viability: 45.9%, IC₅₀ = 17.95 µM). None of the compounds showed toxicity in normal colon cells (CCD‐18Co). qRT‐PCR revealed upregulation of apoptotic (BAX, p53, Caspase‐3/8/9) and antioxidant (SOD‐1/2, CAT, GSS) genes, notably by **3a** in DLD‐1 and **3d** in HT‐29, while **3c** reduced BCL‐2 in HT‐29 cells. ELISA confirmed strong antioxidant induction (**3a**: 70% in DLD‐1) and HDAC inhibition (**3d**: 69% in HT‐29). Western blot showed **3a** increased p38/MAPK expression sevenfold in DLD‐1 and fourfold in HT‐29, while decreasing ERK1. Overall, **3c** and **3d** emerged as the most promising candidates, combining cytotoxic, antioxidant, HDAC inhibitory, and apoptotic effects, and may act as selective therapeutic agents by targeting the p38/MAPK–ERK1 pathway in colorectal cancer.

## Graphical abstract

New sulfonamide‐Schiff bases were synthesized, characterized, and investigated for their cytotoxic, antioxidant, HDAC, and apoptotic activities in human colon cancer cells (DLD‐1 and HT‐29). Compounds **3c** and **3d** emerged as promising candidates to act as selective therapeutic agents by targeting the p38/MAPK‐ERK1 pathway in colorectal cancer.

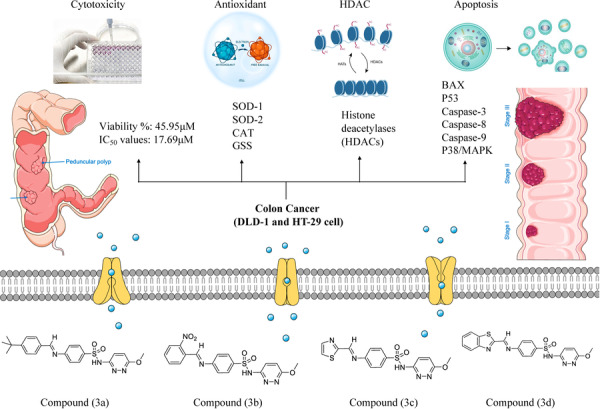

**Abbreviations**
BAX: Bcl‐2‐associated X protein
BCL10: B‐cell lymphoma/leukemia 10
c‐Myc: cellular myelocytomatosis
Caspases: cysteine‐aspartic proteases
CAT: catalase
DPPH: 2,2‐diphenyl‐1‐picrylhydrazyl
DTT: dithiothreitol
Tris: Tris(hydroxymethyl)aminomethane
EGFR: epidermal growth factor receptor
ELISA: enzyme‐Linked immunosorbent assay
ERK1: extracellular signal‐regulated kinase 1
FGF20: fibroblast growth factor 20
GADD45A: growth arrest and DNA‐damage‐inducible protein GADD45 alpha
GSS: glutathione synthetase
HDAC: histone deacetylases
IC_50_
: half‐maximal inhibitory concentration
mTOR: mammalian target of rapamycin
MTT: 3‐(4,5‐dimethylthiazol‐2‐yl)‐2,5‐diphenyl tetrazolium bromide
p38/MAPK: p38 mitogen‐activated protein kinases
p53: tumor protein P53
PARP: poly‐ADP ribose polymerase
PI3K: phosphatidylinositol‐3 kinase
RIPK2: receptor‐interacting protein kinase 2
SALL4: sal‐like protein 4
SDS: sodium dodecyl sulfate
SOD‐1: superoxide dismutase type 1
SOD‐2: superoxide dismutase type 2
SMP: sulfamethoxypyridazine
TAS: total antioxidant status
TBST: tris buffered saline with tween
TNF: tumor necrosis factor
TNFRSF10B: tumor necrosis factor receptor superfamily member 10B
TNFRSF1A (TNF‐R1): tumor necrosis factor receptor superfamily member 1A
TOS: total oxidant status
VEGFR‐2: vascular endothelial growth factor receptor 2
Wnt: wingless‐related integration site
WST‐8: 2‐(2‐Methoxy‐4‐Nitrophenyl)‐3‐(4‐Nitrophenyl)‐5‐(2,4‐Disulfophenyl)‐2H tetrazolium sodium salt

## 1 Introduction

Cancer is a multifaceted disease that affects the apoptosis mechanism [1] due to uncontrolled cell growth and proliferation [2, 3] associated with HDAC and antioxidant activities [4, 5]. Apoptosis plays a pivotal role in eliminating damaged or abnormal cells, including cancer cells, thereby impeding the uncontrolled growth and dissemination of tumors [2, 6].

Furthermore, the interplay between apoptosis and other cellular processes such as cytotoxicity [7], antioxidant activity [8], and histone deacetylase (HDAC) inhibition plays a pivotal role in cancer biology [9, 10]. Moreover, the modulation of HDAC activity has emerged as a promising approach in cancer therapy, as HDAC inhibitors can induce apoptosis in cancer cells by altering gene expression patterns and chromatin structure [4, 9, 10]. Additionally, compounds such as lutein have demonstrated the ability to induce apoptosis in gastric cancer cells through the generation of reactive oxygen species (ROS), highlighting the connection between antioxidant activity and apoptosis in cancer treatment [8, 11]. Targeting key signaling pathways involved in cell survival, such as the p38/MAPK and ERK1 pathway, has been demonstrated to promote apoptosis and inhibit proliferation in cancer cells, highlighting the therapeutic potential of modulating these pathways [12, 13, 14].

In the realm of cancer treatment, triggering apoptosis in cancer cells is a fundamental strategy to eradicate tumor cells and impede cancer progression. Numerous studies have underscored the importance of apoptosis in the response of cancer cells to diverse treatment modalities, such as chemotherapy and targeted therapies [15, 16, 17, 18, 19].

Research on sulfonamides and Schiff base compounds has illuminated their potential as anticancer agents, antioxidants, HDAC, apoptosis, antibacterial, antiviral, and anti‐inflammatory inhibitors of various biological targets [20, 21, 22, 23, 24, 25, 26, 27, 28, 29, 30, 31]. Therefore, while bringing together two notable motifs, sulfonamide and Schiff base, it was aimed to evaluate the structure–activity relationship by also including groups herewith almost proprietary anticancer activities (tert‐butyl, nitro, thiazole, and benzothiazole) in this synergy. Moreover, the molecular hybridization strategy (designing hybrid molecules with various functionalities), which has been widely accepted in drug chemistry in recent years, has accepted this general perspective as a common goal [32]. To elucidate the complex mechanisms of anticancer activities, understanding its interaction with cytotoxic, antioxidant, and HDAC activities is essential to design effective compounds to induce cells to undergo apoptosis. By targeting these interconnected pathways, our research aims to induce apoptosis in cancer cells, thereby restricting tumor growth.

## 2 Results and Discussion

### 2.1 Chemistry

Sulfonamides are a significant group of medications that encompass a range of pharmacological agents with antibacterial, anti‐carbonic anhydrase, diuretic, hypoglycemic, and antithyroid properties [33]. Numerous sulfonamide derivatives have been reported to exhibit in vitro and in vivo antitumor activity. Molecules have an aromatic/heterocyclic or amino acid sulfonamide chemical skeleton, and their antitumor effects are also explained by various mechanisms. These are carbonic anhydrase inhibition, cell‐cycle disruption in the G1 phase, disruption of microtubule assembly, functional suppression, activator NF‐Y, and angiogenesis (matrix metalloproteinase, MMP) inhibition [34].

**Scheme 1:**
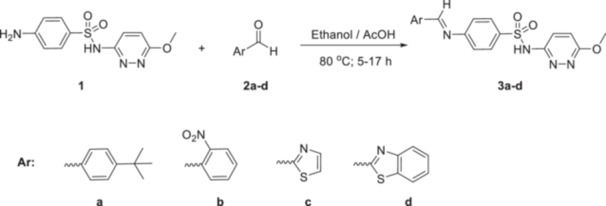
Synthesis of sulfa‐based Schiff bases.

Sulfamethoxypyridazine (**1**), a sulfonamide compound, and four different types of aldehydes (**2a–d**) were reacted for the synthesis of both novel and bioactive sulfonamide‐based Schiff bases (**3a–d**) (Scheme 1). While determining the aldehydes, aldehydes carrying both aromatic (with electron‐releasing and withdrawing substituents) and heterocyclic aromatic (mono and bicyclic) rings were specially selected to investigate the structure's effect on bioactivity. As a result of the studies, ideal reaction conditions were determined. The reactions were carried out using 1.2 equivalents of aldehyde to 1 mole of sulfonamide compound in ethanol with a catalytic amount of acetic acid and at reflux conditions. Reaction times vary from 5 to 17 h. In the first stage of structural characterization, TLC, melting point determination, and FTIR spectroscopy methods were applied. Subsequently, NMR (^1^H and ^13^C/APT), UV‐Vis spectroscopic methods, and LC‐MS/TOF were employed to ascertain the exact structures of the newly synthesized molecules (Supporting Information S1: Figures S2–S18).

All compounds gave [M + H]^+^ ion peaks calculated according to their molecular formulas with high agreement in their LC/MS‐TOF mass spectra: While [M + H]^+^ ion peaks of the compounds were calculated as *m/z* 425.1642, 414.0867, 376.0533, and *m/z* 426.0689; 425.1614, 414.0855, 376.0534, and 426.0673 values were observed in their mass spectra (for **3a–d**, respectively).

When the FTIR data were examined, it was observed that the double‐dentate intense peaks at 3479 and 3387 cm^–1^ belonging to the amino (–NH_2_) group of sulfamethoxypyridazine (**1**) disappeared in the spectrum of the products and were replaced by single‐dentate –NH stretching peaks at 3217, 3340, and 3232 cm^–1^ (**3a–d**, respectively). Although the –NH stretching band was not observed for compound **3c**, other spectroscopic data of the compound revealed the presence of the –NH group. In addition, the aldehyde hydrogen HC═O and aldehyde carbonyl C═O stretching peaks belonging to the starting aldehydes disappeared because of the formation of the products. Instead, it was seen that the stretching belonging to the azomethine (HC═N) group appeared at 1635, 1643, 1625, and 1643 cm^–1^ (**3a–d**, respectively) [35].

According to the ^1^H NMR signals of the compounds, the one‐proton singlet peaks at 8.58, 8.88, 8.83, and 8.98 ppm belong to the N═CH azomethine group; the one‐proton singlet peaks at 5.95 for **3a–c** and 7.77 ppm for **3d** belong to the NH structure attached to the sulfonyl group (SO_2_–NH) (**3a–d**, respectively). These two peaks observed in the ^1^H NMR spectra of the compounds are strong evidence for the formation of the compounds [36]. The three‐proton singlet peak at 3.85 ppm in **3d** and 3.84 ppm in **3a–c** compounds belongs to the methoxy (OCH_3_) protons carried by the sulfonamide skeleton. The signals between 6.17 and 8.16 ppm are the chemical shifts of the aromatic ring protons with different chemical environments in the products.

One of the distinctive peaks in the ^13^C NMR/APT spectra of the compounds is the quaternary carbon signal belonging to the azomethine (N═CH) group [37]. These peaks were observed at 158.36, 159.67, 156.12, and 167.03 ppm for compounds **3a–d**, respectively. The methyl (CH_3_) carbon of the methoxy group from the sulfamethoxypyridazine part in the products was observed at chemical shift values of 54.80, 54.80, 54.81, and 55.04 ppm for compounds **3a–d**, respectively. The peaks detected at 121.82–166.55 ppm were ascribed to the carbons of aromatic rings exhibiting distinct chemical environments.

The conjugated molecular structure of the compounds led us to conduct UV–Vis studies. The investigation of UV–Vis spectra of the new molecules revealed that they displayed maximum absorbances (*λ*_max_) in the range corresponding to the wavelengths of 200–204, 242–260, and 312–353 nm. These λ_max_ values are compatible with the structures of the molecules. The bands observed between 200 and 204 and 242–260 nm correspond to the E1 and B bands (**3a–d**, respectively) originating from the π–π* transition of the aromatic chromophore. The bands observed between 312 and 353 nm originate from the n–π* transitions of the heteroatoms containing unshared electron pairs (n electrons) in the molecules [38].

### 2.2 Biological Activity

#### 2.2.1 Cytotoxicity of Sulfonamide‐Derived Schiff Bases in Colorectal Cancer (DLD‐1, HT‐29) and Normal Colon (CCD‐18Co) Cells Assessed by MTT and WST‐8

**Figure 1:**
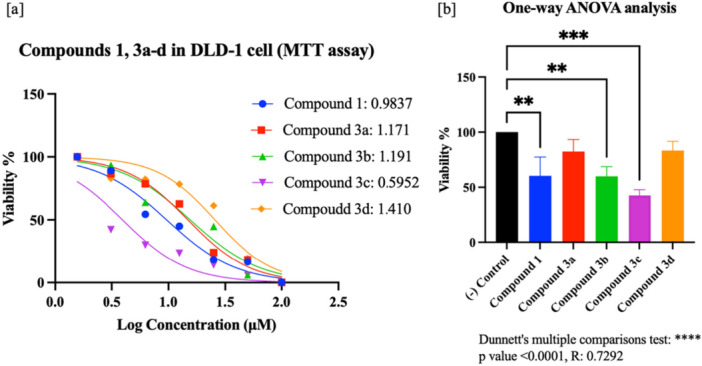
(a) Cell viability (%) and half‐maximal inhibitory concentration (LogIC₅₀) for compound **1** and **3a–d** in DLD‐1 cells (MTT assay); (b) one‐way ANOVA (Dunnett's multiple comparisons) of percentage viability for each compound (dose: 100 µM), shown as bar graphs relative to control (cells + medium) (n: 3).

**Figure 2:**
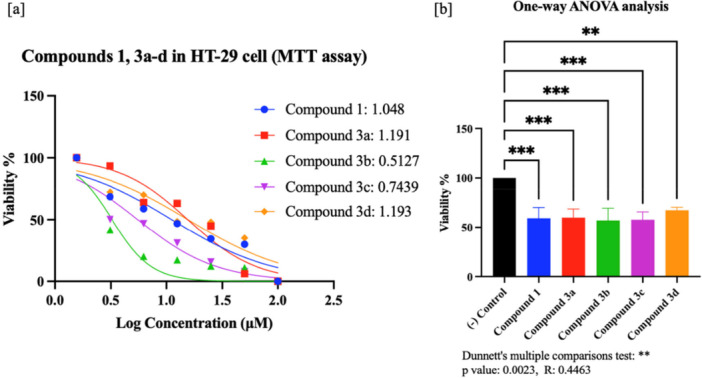
(a) Cell viability (%) and half‐maximal inhibitory concentration (LogIC₅₀) for compound **1** and **3a–d** in HT‐29 cells (MTT assay); (b) one‐way ANOVA (Dunnett's multiple comparisons) of percentage viability for each compound (dose: 100 µM), displayed as bar graphs relative to control (cells + medium) (n: 3).

In this study, compound **1** and its sulfonamide–Schiff base derivatives (**3a–d**) were evaluated for in vitro cytotoxicity in DLD‐1 and HT‐29 colorectal cancer cells and in the normal colon cell line CCD‐18Co using MTT and WST‐8 assays. Percentage viability and IC₅₀ values were determined (Supporting Information S1: Tables S1–S5), and representative dose–response curves with statistical analyses (*p* < 0.0001) are provided in Figures 1 and 2. Across the tested ranges (MTT, 1.56–100 μM; WST‐8, 6.25–100 μM), all compounds significantly reduced viability in both cancer cell lines, with **3b** being most effective in HT‐29 and **3c** in DLD‐1. Importantly, no appreciable toxicity was observed in CCD‐18Co (Supporting Information S1: Table S3).

Prior work has reported the cytotoxic and antiproliferative properties of sulfonamide‐derived Schiff bases across multiple cancer models. For example, Aydın et al. demonstrated robust MTT‐based cytotoxicity in lung cancer cells [27], and studies in ovarian (A‐2780), breast (MCF‐7), and colorectal (HCT‐116, HT‐29) lines have shown micromolar IC₅₀ values for this class [39, 40, 41]. These findings support a selective cytotoxic potential of sulfonamide–Schiff bases in tumor cells, a view reinforced by recent reviews highlighting the broad anticancer scope of Schiff bases bearing a sulfonamide moiety [42]. Consistent with this literature, our compounds display strong activity in the colorectal setting—most notably **3b** in HT‐29 (IC₅₀ = 3.26 µM) and **3c** in DLD‐1 (IC₅₀ = 3.94 µM)—underscoring that this chemotype can achieve low‐micromolar potency in colorectal cancer models (Supporting Information S1: Tables S1–S2).

In DLD‐1 cells, MTT results indicated differing degrees of cytotoxicity across the series. The strongest effect was observed with **3c**, which reduced viability to 37.70% at its minimum and yielded an IC₅₀ of 3.94 µM. In contrast, **3d** was the weakest (IC₅₀ = >100 µM). Compounds **1**, **3a**, and **3b** showed intermediate activity (IC₅₀ = 9.63–15.54 µM). Collectively, these findings highlight the pronounced potency of **3c** in DLD‐1 and the unexpectedly limited activity of **3d** (Supporting Information S1: Table S1; Figure 1).

In HT‐29 cells, the most pronounced cytotoxicity was observed with **3b**, which reduced viability to 46.57% at a minimum and achieved an IC₅₀ of 3.26 µM. **3c** showed moderate activity (IC₅₀ = 5.54 µM), whereas the remaining compounds displayed weaker effects with higher IC₅₀ values (11.16–15.58 µM). Notably, **3d**—despite structural features that might be expected to enhance activity—ranked among the least effective in HT‐29. All differences were statistically significant (*p* < 0.0001) (Supporting Information S1: Table S2 and Figure 2).

In the normal colon line CCD‐18Co, percentage viability remained **>**88% for all compounds, and no appreciable cytotoxicity was detected (Supporting Information S1: Table S3).

**Figure 3:**
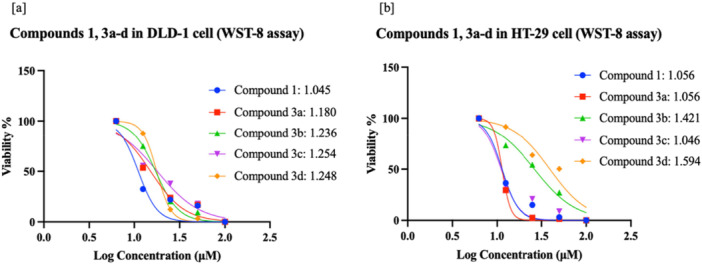
(a) Cell viability (%) and half‐maximal inhibitory concentration (LogIC₅₀) for compound **1** and **3a–d** in DLD‐1 cells by WST‐8 assay; (b) cell viability (%) and LogIC₅₀ for compound **1** and **3a–d** in HT‐29 cells by WST‐8 assay (Control: cells with medium only) (n: 3).

WST‐8 results revealed some assay‐dependent differences relative to MTT. In DLD‐1, the largest drop in viability was seen with **3c** (45.95% at minimum), yet IC₅₀ values were comparable for **3a** (>100 μM) and **3c** (17.95 μM). In HT‐29, **1** (min viability 62.01%) stood out, whereas **3a, 3b, 3c** and **3d** were less active min viability 63.01–75.17% and IC_50_: >100 µM. (Supporting Information S1: Tables S4–S5; Figure 3).

The selectivity index (SI = IC₅₀normal/IC₅₀cancer) was calculated using CCD‐18Co cells as the normal control. Since all compounds exhibited IC₅₀ values > 100 µM in CCD‐18Co cells, the reported SI values represent conservative lower bounds. The results demonstrated that compounds **3c** and **3b** exhibited particularly strong therapeutic selectivity, with SI values > 25.4 (DLD‐1) and > 30.7 (HT‐29), respectively. Importantly, compound **3d** also showed favorable selectivity with SI values > 3.9 in DLD‐1 and > 6.4 in HT‐29, highlighting its translational potential as an additional lead candidate. Compound **1** showed moderate selectivity (SI > 9–10), while **3a** remained above the clinically relevant SI threshold of 2. Collectively, these findings emphasize that **3b**, **3c**, and **3d** are the most promising molecules in the series, offering a favorable therapeutic window for colorectal cancer treatment (Supporting Information S1: Table S6).

Taken together, the MTT and WST‐8 data indicate that the cytotoxic profiles of sulfonamide–Schiff bases are cell‐type dependent and shaped by substitution patterns. Notably, **3c** is more potent in DLD‐1, whereas **3b** dominates in HT‐29, consistent with the selective engagement of biological targets by different substituents in distinct cellular contexts [27, 39]. Direct colorectal cancer reports that combine the identical sulfonamide–Schiff base core with 2‐nitro or thiazole are limited; however, the closest analogs align with our trends. For example, thiazole‐ plus sulfonamide‐based Schiff bases formulated as 5‐FU co‐crystals achieved 100% inhibition at 100 µg/mL by MTT in SW‐480 [43]. Likewise, Schiff bases bearing a 2‐nitro (5‐nitrosalicylidene) motif showed high potency at ~1 µM as Pd(II) complexes in HeLa/MCF‐7, underscoring the 5‐nitrosalicylidene scaffold as a productive electronic/interaction motif; and several Schiff‐base complexes display meaningful antiproliferative activity in Caco‐2 (e.g., Pd(II) IC₅₀ ≈ 16.6 µg/mL) [44]. Collectively, although exact colorectal cancer exemplars with our full structural combination are scarce, closely related pharmacophores/scaffolds exhibit sub‐ to low‐micromolar efficacy in colon models, supporting the strong DLD‐1 profile of **3c** and the cell‐type‐selective activity of **3b** and indicating that our results are biologically plausible and literature‐consistent [45, 46, 47, 48, 49, 50, 51, 52].

By contrast, **3d** (benzothiazole) was weaker in both assays. A comparable scenario has been attributed to reduced coordination flexibility (structural rigidity) of benzothiazole‐containing bidentate Schiff bases in metal complexes and to increased aromaticity diminishing productive target interactions—together limiting cytotoxicity [53]. Consistent reports also note that benzoazole Schiff bases can show lower permeability/affinity in certain cell lines, yielding attenuated cytotoxicity [40, 41]. Moreover, permeability/bioavailability can vary across lines as a function of lipophilicity, charge state, and dipole moment; for instance, a nano‐Schiff base complex with higher lipophilicity/dipole demonstrated facilitated membrane passage [54]. Finally, the absence of appreciable toxicity in CCD‐18Co supports a degree of selectivity for malignant cells. Overall, these data indicate that biological responses of sulfonamide–Schiff bases depend on both structure and cell type and that structural differences can steer selectivity across colorectal models [42].

#### 2.2.2 qRT‐PCR Analysis of Antioxidant and Apoptotic Genes in Colorectal Cancer Cell Lines

In DLD‐1 cells, all compounds elicited a clear apoptotic response, increasing the transcripts of BAX, p53, and caspases. The BAX/BCL‐2 ratio was maximal with **3a** (~7.2‐fold) and **3b** (~9‐fold), consistent with robust activation of mitochondria‐mediated apoptosis. Although **3c** elevated apoptotic genes, it did not produce a meaningful change in GSS, an indicator of antioxidant response. By contrast, **1**, **3a**, **3b**, and **3d** increased GSS together with SOD‐1/2 and CAT, reflecting an adaptive redox‐balancing response (Table 1).

**Table 1:** qRT‐PCR analysis of apoptotic and antioxidant gene expression changes (up‐/downregulation) induced by sulfonamide‐derived Schiff bases in DLD‐1 cells.

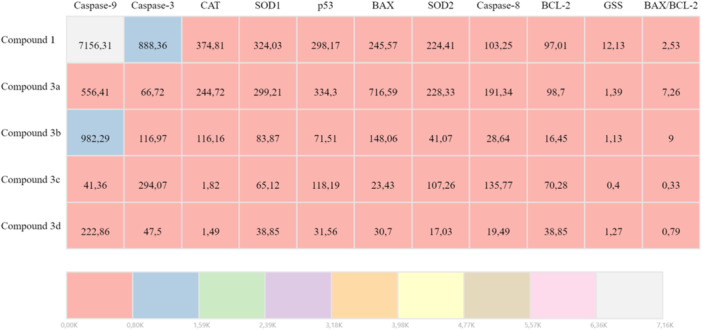

In HT‐29 cells, all compounds produced a pronounced increase in apoptosis‐related genes: p53, CASP8, and CASP9 rose robustly across the series, while BAX increased for all except **3c**. The BAX/BCL‐2 ratio was highest with **3a** (~2.4‐fold), **3b** (~1.5‐fold), and **3d** (~1.4‐fold) showed more moderate rises. For the antioxidant response, SOD‐1/2 and CAT increased with all compounds, and GSS increased except with **3c**. Together, these results indicate that **3a** most strongly triggers the apoptotic program in HT‐29, whereas **3c** is comparatively weaker in this line (Table 2).

**Table 2:** qRT‐PCR analysis of apoptotic and antioxidant gene expression changes (up‐/down‐regulation) induced by sulfonamide‐derived Schiff bases in HT‐29 cells.

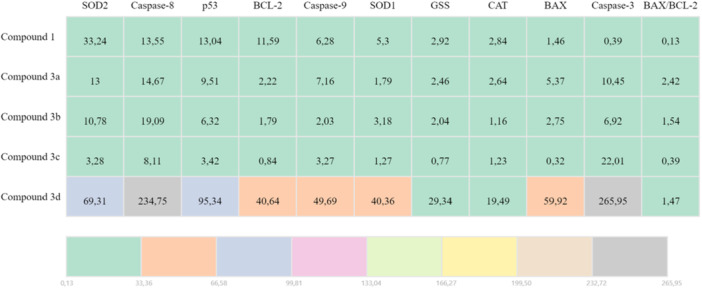

Reports across multiple cancer models indicate that sulfonamide‐derived Schiff bases can modulate apoptotic pathways. For example, several compounds suppress FGF20 and SALL4 expression, thereby inhibiting the Wnt/β‐catenin axis and restraining cell proliferation [55]. In pancreatic and colorectal cancer cells, this chemotype has been shown to induce cell death by upregulating apoptotic genes such as BAX, BAK, CASP3/9, and p53 [40, 56]. Likewise, in lung and colorectal models, downregulation of EGFR, mTOR, and PI3K—together with increased p53—supports an apoptosis‐promoting effect of sulfonamide Schiff bases [41]. Collectively, these observations indicate that this class can trigger programmed cell death via multiple cellular routes, in agreement with the signatures observed in our study.

In colorectal models, 2,4‐di‐*tert*‐butylphenol (2,4‐DTBP) suppresses the anti‐apoptotic proteins BCL‐2 and Survivin and thereby enhances apoptosis in HCT‐116. Although demonstrated at the protein level, this supports the notion that tert‐butyl phenolics can strengthen the pro‐apoptotic axis in colorectal cancer cells [45]. Moreover, p38/MAPK activation is frequently associated with apoptosis induction in colorectal cancer cells. The pro‐apoptotic role of p38 is documented both in reviews and by direct experimental evidence in HT‐29 [57, 58]. Consistent with this framework, our compound **3a** elicited robust qRT‐PCR signatures—BAX/BCL‐2 ~ 2.4‐fold in HT‐29 and ~7.2‐fold in DLD‐1—indicating potent engagement of mitochondria‐mediated apoptosis in both lines.

Nitro–cap designs based on 3‐nitro‐2*H*‐chromene engage HDAC1/2 with nanomolar potency and have shown antiproliferative effects exceeding SAHA/MS‐275 across multiple cell lines [51]. In colorectal cancer models, pan‐HDAC inhibition is linked to strong growth suppression and apoptosis; a recent pan‐HDAC inhibitor exhibited high sensitivity in HCT‐116 and HT‐29 [50], and HDAC blockade in colorectal cancer cells also downregulates the EGFR/ERK axis [59]. In line with this template, our data show that **3b** produces a ~ ninefold increase in BAX/BCL‐2 in DLD‐1 and a moderate (~1.5‐fold) increase in HT‐29, consistent with an electron‐withdrawing, properly positioned nitro group promoting engagement of epigenetic/apoptotic pathways.

While **3c** strongly reduced viability in DLD‐1 (MTT IC₅₀ = 3.94 µM), GSS induction was limited, and BAX did not increase in HT‐29. Two rationales commonly invoked in the literature explain this kind of “viability↓ but early transcript signature weak” divergence: (*i*) *Timing/readout mismatch*: Viability loss can initiate via early cell‐cycle perturbations or caspase‐independent routes (e.g., mitotic/ER stress, AIF pathways), with the apoptotic transcript signature emerging later. In DLD‐1/HT‐29, a sulfonamide derivative (MM131) showed a similar dynamic with S‐phase/G2–M accumulation and caspase activation [60]. (*ii*) *Epigenetic feedback*: The +33% HDAC activity observed for **3c** in DLD‐1 may reflect a compensatory epigenetic response rather than direct target inhibition per se. Together, these mechanisms can reconcile **3c**'s early signaling effects (p38 ×5 in DLD‐1) and strong viability loss with a comparatively modest qRT‐PCR window.

The limited efficacy of **3d** is consistent with a developability trade‐off frequently emphasized in the literature: increased aromatic rigidity/lipophilicity can compromise solubility and passive permeability, thereby attenuating measured biological effects (aromaticity ↑ /lipophilicity↑ → solubility & permeability↓ → weaker activity) [61, 62]. Additionally, cell‐line–specific bioavailability differences (e.g., HT‐29 vs. DLD‐1) may further contribute.

#### 2.2.3 ELISA Analysis of Antioxidant and HDAC Activity, and Western Blot Analysis of Apoptotic Signaling

**Figure 4:**
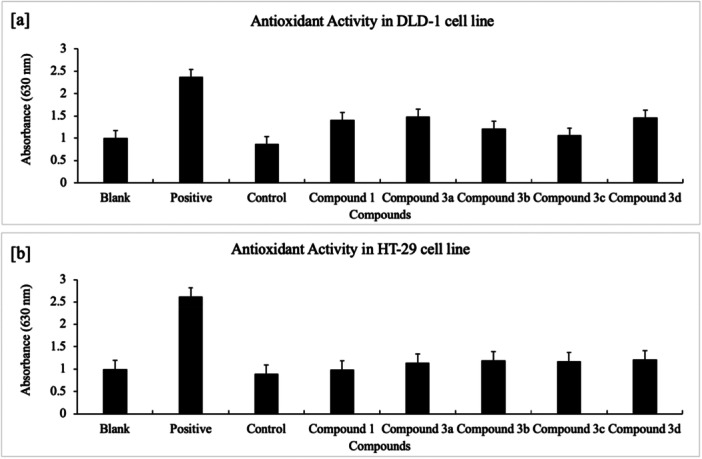
(a) ELISA‐based induction of total antioxidant capacity for compound **1** and **3a–d** in DLD‐1 cells; (b) ELISA‐based induction for compound **1** and **3a–d** in HT‐29 cells (Blank: ddH₂O; Positive control: Trolox; Control: cells + medium).

In parallel with the cytotoxic and qRT‐PCR antioxidant/apoptosis signatures, ELISA‐based measurements of total antioxidant capacity (TAC) showed induction by all compounds, more prominently in DLD‐1. The highest increase was observed with **3a** (+70%, MTT IC₅₀ = >100 µM), whereas in HT‐29 the top induction was obtained with **3d** (+35%, MTT IC₅₀ = >100µM) (Figure 4; Tables 1, 2). This pattern aligns with the qRT‐PCR increases in SOD‐1/2 and CAT (with GSS elevated for most compounds), indicating a coordinated redox re‐equilibration.

**Figure 5:**
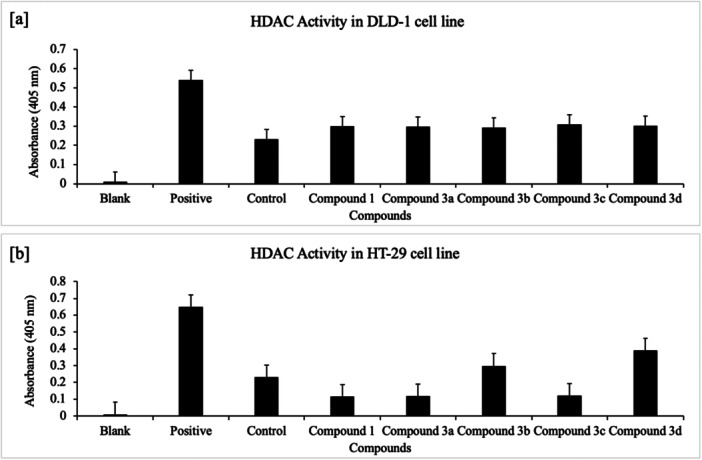
(a) ELISA‐based induction of HDAC activity for compound **1** and **3a–d** in DLD‐1 cells; (b) ELISA‐based induction of HDAC activity for compound **1** and **3a–d** in HT‐29 cells (Blank: ddH₂O; Positive control: HeLa nuclear extract; Control: cells + medium).

Similarly, the target‐directed epigenetic readout HDAC activity (ELISA) increased for all compounds in DLD‐1, peaking with **3c** (+33%, MTT IC₅₀ = 3.94 µM). In HT‐29, a marked increase was observed with **3d** (+69%, MTT IC₅₀ = >100 µM) and a moderate increase with **3b** (+29%, MTT IC₅₀ = 3.26 µM), whereas **1/3a/3c** showed no significant induction (Figure 5). This contrast supports that the magnitude of HDAC modulation depends on the scaffold and cell line. Notably, the 2‐nitro substitution present in **3c** fits an SAR pattern in which 3‐nitro‐2*H*‐chromene “cap” motifs enable nanomolar interactions with HDAC1/2 [51].

**Figure 6:**
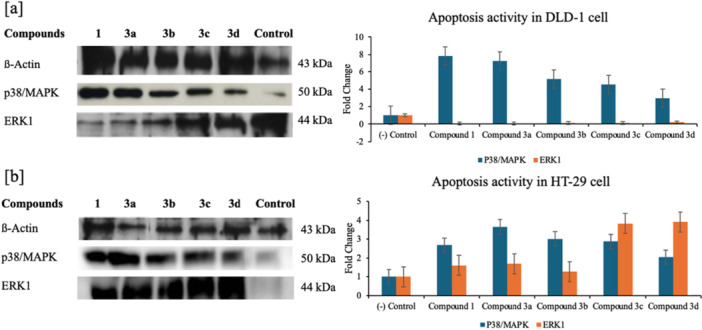
(a) Western blot analysis of p38/MAPK and ERK1 protein levels in DLD‐1 cells treated with compound **1** and **3a–d**; (b) corresponding analysis in HT‐29 cells (Housekeeping: β‐actin; Control: cells + medium).

At the signaling level, Western blot analyses showed apoptosis‐associated p38/MAPK activation most prominently in HT‐29 with **3a** (×4; MTT IC₅₀ = 15.54 µM) and **3b** (×3; MTT IC₅₀ = 3.26 µM), while in DLD‐1, the highest p‐p38 increase occurred with compound **1** (×8; MTT IC₅₀ = 9.63 µM), followed by **3a** (×7) (Figure 6). ERK1 signaling declined preferentially in DLD‐1, with the strongest reduction observed for **3d** (0.8×); no meaningful decrease was detected in HT‐29. These signaling profiles are consistent with the qRT‐PCR signatures (BAX↑/BCL‐2↓, p53, and caspases↑) and indicate cell type– and scaffold–dependent progression of effects.

Sulfonamide‐derived Schiff bases have been shown in diverse models to enhance antioxidant/ROS responses and to trigger apoptosis via caspase activation [56, 63, 64, 65]. Along the HDAC axis, sulfonamide/Schiff‐base–like designs can engage HDAC1/2/3/4, and colorectal cell lines exhibit high sensitivity to HDAC interventions [50, 51, 63, 64, 66, 67, 68]. Regarding p38/ERK, the link between p38 activation and apoptosis in colon cells—and p38‐dependent apoptotic responses specifically in HT‐29—is well documented [40, 41, 46, 47, 48, 56].

The *tert*‐butyl phenolic motif in **3a** is known to confer strong antioxidant capacity through free‐radical trapping/phenoxyl‐radical stabilization, and 2,4‐di‐*tert*‐butylphenol has been reported to induce apoptosis in colorectal cancer by suppressing the BCL‐2/Survivin axis [45, 69]. Examples of p38‐mediated apoptosis in HT‐29 (e.g., tanshinone I, ursolic acid) are mechanistically consistent with our **3a/**HT‐29 p‐p38↑ profile [46, 47, 48].

As noted, 3‐nitro‐2*H*‐chromene cap designs engage HDAC1/2 with nanomolar potency and have produced antiproliferative effects surpassing SAHA/MS‐275 across multiple lines [51]. In colorectal cell models, pan‐HDAC targeting drives robust growth inhibition and apoptosis, with high sensitivity in HT‐29/HCT‐116 [50]. Within this framework, the composite signature of **3b**—BAX/BCL‐2↑ (~9×) in DLD‐1 and HDAC elevation (~+29%) in HT‐29—is coherent, reflecting an electron‐withdrawing, properly positioned nitro group that promotes engagement of epigenetic/apoptotic pathways.

In HT‐29, apoptosis induction via p38 phosphorylation by phenolic structures (e.g., tanshinone I and related natural products) is well documented and is mechanistically consistent with the p‐p38↑ profiles observed for **3a/3b** in this line [46, 47, 70]. Recent reviews summarizing the impact of natural products on the MAPK/p38 pathway in colorectal cancer further support this connection [46, 47, 57].

Despite **3c** producing HDAC + 33% and p38 ×5 activation in DLD‐1 (with strong viability loss but limited GSS by qRT‐PCR), a readout‐timing/endpoint effect is plausible: Early cell‐cycle/ER‐stress perturbations and/or caspase‐independent routes (e.g., AIF) may rapidly reduce viability, while the apoptotic transcript signature becomes prominent later [60]. Additionally, HDAC‐axis feedback in colorectal cells (including isoform‐selective changes) can register as an overall increase in ELISA yet appear asynchronous with qRT‐PCR readouts [50, 59]. Together, these factors rationalize why **3c** appears potent in MTT/WST‐8 yet more moderate in the ELISA/Western‐blot/qRT‐PCR segment of this section.

Although **3d** achieved a + 69% increase in HDAC activity in HT‐29, its effects at the p38/ERK endpoints were limited. This pattern is consonant with a widely noted developability trade‐off: Aromaticity ↑ /lipophilicity↑ → solubility & passive permeability↓ → attenuated biological effect [71, 72]. Cell‐type–specific uptake/distribution (as influenced by lipophilicity, dipole, and charge state) may further contribute [71, 73]. Reports of decreased p‐p38/ERK with benzothiazole scaffolds also align with our observation [49]. At the same time, benzothiazole–hydroxamic acid derivatives can interact strongly with HDAC1/3/4, and HT‐29 is generally sensitive along the HDAC axis [50, 52, 74, 75]. Conversely, the benzothiazole core has been associated with multifunctional antioxidant/ROS modulation, offering an additional—though context‐dependent—mechanistic dimension [45, 71, 76].

## 3 Conclusion

Effective results were obtained for compound **1** and sulfonamide derivative Schiff bases (**3a–d**) in their in vitro anticancer activities on DLD‐1 and HT‐29 colon cancer cell lines.

The most effective compounds are **3c** (Viability %: 37.70, IC_50_ values: 3.94 μM) in the DLD‐1 cell line, and compound **3b** (target) (Viability %: 46.57, IC_50_ values: 3.26 μM) in the HT‐29 cell line by MTT assay. Also, compounds (**1**, **3a–d**) did not cause toxic effects on normal colon cells (CCD‐18Co). The most effective compounds are **3c** (target) **(**viability %: 45.95, IC_50_ values: 17.95 μM) in the DLD‐1 cell line, and compound **3a** (viability %: 63.01, IC_50_ values: >100 μM) (target) in the HT‐29 cell line by WST‐8 assay.

mRNA expression levels of BAX (**3a**), p53 (**3a**), Caspase‐3 (**3c**), Caspase‐8 (**3a**), and Caspase‐9 (**3b**), the genes related to apoptotic activity, and SOD‐1 (**3a**), SOD‐2 (**3a**), CAT (**3a)**, and GSS (**3a**), the genes related to antioxidant activity, showed a remarkably high increase in compounds in DLD‐1 cell line. mRNA expression levels of BAX (**3d**), p53 (**3d**), Caspase‐3 (**3d**), Caspase‐8 (**3d**), and Caspase‐9 (**3d**), the genes related to apoptotic activity, and SOD‐1 (**3d**), SOD‐2 (**3d**), CAT (**3d**), and GSS (**3d**), the genes related to antioxidant activity, showed a remarkably high increase in compounds in HT‐29 cell line. mRNA expression levels of BCL‐2 (**3c**) were decreased in the HT‐29 cell line.

The antioxidant induction activity is higher in DLD‐1 cells (**3a**, 70%) compared with HT‐29 cells (**3d,** 35%), and HDAC inhibition activity was highest in HT‐29 cells (**3d**, 69%) and in DLD‐1 cells (**3c**, 33%) by ELISA assay.

The p38/MAPK protein expression levels (sevenfold) increased, and ERK1 protein expression levels (onefold) decreased in compound **3a** compared with the control by western blot analysis in DLD‐1 cells. In addition, the p38/MAPK protein expression levels (fourfold) increased in compound **3a** compared with the control in HT‐29 cells.

Effective cytotoxic, antioxidant, and HDAC activities of the compounds (especially **3c**–**d**) in colon cancer were found, and it was determined that they could be used as a useful agent in cell death by affecting the p38/MAPK, ERK1 anticancer pathway.

Many therapeutic agent syntheses containing tert‐butyl, nitro, thiazole, and benzothiazole nuclei have been reported in the literature. The biological activities of these groups in different areas have also been generally accepted. Recently, these groups have been used in the design of hybrid molecules that are specific to various anti‐tumor receptors by combining them with different functional groups with high bio‐potential. All efforts are to offer new possibilities for clinical treatments. Therefore, the aim is to develop drugs that are pioneering, selective, free from unwanted side effects, and less likely to show cross‐resistance against cancer by using compounds containing these groups, which are constantly reported to be pharmacologically active. Moreover, these compounds could be used as a potential source of cancer therapy via the pharmaceutical industry.

In summary, sulfonamide‐derived Schiff bases (particularly **3c** and **3d**) demonstrated selective anticancer potential by inducing apoptosis, enhancing antioxidant responses, inhibiting HDAC activity, and modulating p38/MAPK–ERK1 signaling in colon cancer cells while exhibiting no toxicity in normal colon cells. These findings highlight the novelty of combining sulfonamide and Schiff base scaffolds with bioactive substituents as promising lead structures for colorectal cancer therapy. Future studies will focus on validating these effects in in vivo colorectal cancer models, investigating pharmacokinetic and toxicity profiles, and performing rational structural modifications to improve potency and selectivity. Such efforts will pave the way for the preclinical development of innovative anticancer agents derived from this compound series.

## 4 Experimental

### 4.1 Chemistry

#### 4.1.1 General

The reagents and solvents were purchased from Merck (Darmstadt, Germany) or Sigma‐Aldrich (St. Louis, MO, USA) and used without further purification. All melting points (mp) were measured in open glass capillaries in the Electrothermal IA9200 mp apparatus (Staffordshire, UK) and were uncorrected. All reaction progresses were monitored by thin‐layer chromatography (TLC) using 20 × 20 aluminum sheets from Merck (TLC Silica gel 60 F_254_). A Shimadzu Affinity‐1 FTIR spectrophotometer (Duisburg, F.R. Germany) was used to record IR spectra using the attenuated total reflectance (ATR) technique in the range of 4000–600 cm^–1^ at a resolution of 8 cm^–^^1^. ^1^H and APT ^13^C NMR spectral data were recorded with a Varian Mercury‐400 (Seattle, WA, USA) in DMSO‐*d*_6_, as a solvent. Chemical shifts (*δ*) are reported in parts per million (ppm) and coupling constants (*J*) are reported in Hertz (Hz). Multiplicity was assigned as s (singlet), d (doublet), t (triplet), and m (multiplet). High‐resolution mass spectra (HRMS) were measured on an Agilent LC/MS‐QTOF spectrometer (Santa Clara, USA) (Supporting Information S1: Figure S2–S18).

#### 4.1.2 General Procedure for the Synthesis of Sulfonamide Derivative Schiff Bases **3a–d**

A catalytic amount of glacial acetic acid was added to a mixture of sulfonamide 1 (100 mg, 0.36 mmol) and aldehydes **2a–d** (0.432 mmol, 1.2 eq.) in 7 mL of absolute ethanol. The reaction mixture was refluxed, and during this time, the starting material status and product formation in the reactions were monitored by TLC. The solvent of the reaction mixtures that were finished at appropriate reaction times was removed in a rotary evaporator. The solid crude product was washed with water to free the catalyst from acetic acid and dried. It was then washed successively with cold methanol (3 × 10 mL) and cold acetonitrile (3 × 10 mL). Some crude products obtained (**3a**, **d**) were subjected to recrystallization for further purification, while some (**3b**, **c**) were subjected to column and preparative chromatography methods.

Sulfamethoxypyridazine (**1**) was purchased commercially, and no purification was performed before the study.

4‐{[4‐(*tert*‐Butyl)benzylidene]amino}‐*N*‐(6‐methoxypyridazine‐3‐yl)benzene sulfonamide (C_22_H_24_N_4_O_3_S) (**3a**): Recrystallization from acetonitrile‐THF. White solid. Yield: 35%. Mp: 209°C–210°C. Rf: 0.63 (2:1 ethyl acetate/hexanes). UV‐Vis (THF, c: 2.36 × 10^–4^ mol L^–^^1^): *λ*_max_ (ɛ, L/mol. cm) 204 (7131), 242 (11 766), 315 (15 610). FTIR (ATR, cm^–1^): ν 3217 (–NH stretching, w), 3132–3008 (Ar –CH stretching, w), 2962 and 2870 (Aliph –CH stretching, w), 1635 (C═N stretching, m), 1573 and 1465 (Ar C═C stretching, m), 1388 (–SO_2_ asym stretching, s), 1296 (C–N stretching, s), 1134 (–SO_2_ sym stretching, s), 1087 (C–O–C sym stretching, s), 1002 (1,4‐disubstituted aromatic ring in‐plane CH bending, m), 817 and 725 (1,4‐disubstituted aromatic ring out‐of‐plane CH bending, s). ^1^H NMR (400 MHz, DMSO‐*d*_6_, ppm*) δ* 8.58 (s, 1H, –N═CH), 7.86 (dd, *J*: 11.1, 6.31 Hz, 4H, Ar═CH), 7.57 (d, *J*: 8.4 Hz, 2H, Ar═CH), 7.47 (d, *J*: 8.6 Hz, 1H, Ar═CH), 7.34 (d, *J*: 8.4 Hz, 2H, Ar═CH), 6.56 (d, *J*: 8.7 Hz, 1H, Ar═CH), 5.95 (s, 1H, NH), 3.84 (s, 3H, –OCH_3_), 1.32 (s, 9H, –C(CH_3_)_3_). ^13^C NMR (100 MHz, DMSO‐*d*_6_, ppm) *δ* 158.36 (N═CH), 155.56 (Ar‐C), 153.06 (Ar‐C), 150.20 (Ar‐C), 145.22 (Ar‐C), 140.80 (Ar‐C), 134.38 (Ar‐C), 129.92 (Ar═CHx2), 129.38 (Ar═CHx2), 128.94 (Ar═CHx2), 126.47 (Ar═CHx2), 126.24 (Ar═CH), 121.82 (Ar═CH), 54.80 (–OCH_3_), 35.54 (–C(CH_3_)_3_), 31.24 (–(CH_3_)_3_). HR‐MS Calcd. for C_22_H_24_N_4_O_3_S [M + H]^+^‐ 425.1642; found 425.1614.

*N*‐(6‐Methoxypyridazine‐3‐yl)‐4‐[(2‐nitrobenzylidene)amino]benzene sulfonamide, (C_18_H_15_N_5_O_5_S) (**3b**): Column chromatography from 2:1 ethyl acetate/hexanes followed by preparative layer chromatography in 2:1 ethyl acetate/hexanes (containing 1.5% N(Et)_3_). Pale yellow solid. Yield: 60%. Mp: 193°C–195°C. Rf: 0.60 (2:1 Ethyl acetate: Hexanes). UV‐Vis (THF, c: 2.42 × 10^–4^ mol L^–1^): λ_max_ (ɛ, L/mol. cm) 204 (7131), 242 (11 766), 315 (15 610). FTIR (ATR, cm^–1^): ν 3340 (N–H stretching, w); 3178, 3055 (Ar‐CH stretching, w); 2954, 2870 (Aliph –CH stretching, w); 1643 (C═N stretching, w); 1597, 1527 (Ar C═C stretching, m); 1404 (SO_2_ asym stretching, m); 1134 (SO_2_ sym stretching, s); 1080 (C–O stretching, s); 933 (1,2‐disubstituted aromatic ring out‐of‐plane CH bending, m‐s); 833 (1,4‐disubstituted aromatic ring out‐of‐plane CH bending, m). ^1^H NMR (400 MHz, DMSO‐d_6_, ppm): *δ* 8.88 (s, 1H, N═CH), 8.16 (dd, *J:* 11.3, 7.5 Hz, 2H, Ar═CH), 7.91 (d, *J:* 8.6 Hz, 2H, Ar ═CH), 7.81 (t, *J:* 7.9 Hz, 2H, Ar═CH), 7.47 (d, *J:* 8.6 Hz, 1H, Ar═CH), 7.38 (d, *J:* 8.5 Hz, 2H, Ar═CH), 6.56 (d, *J:* 8.7 Hz, 1H, Ar═CH), 5.95 (s, 1H NH), 3.84 (s, 3H, –OCH_3_). ^13^C NMR/APT (100 MHz, DMSO‐d_6_, ppm): *δ* 165.00 (Ar‐C), 159.67 (N═CH), 153.07 (Ar‐C), 149.86 (Ar‐C), 147.77 (Ar‐C), 138.80 (Ar‐C), 134.72 (Ar═CH), 134.64 (Ar═CH), 134.35 (Ar═CH), 132.81 (Ar═CHx2), 131 (Ar‐C), 130.32 (Ar═CH), 125.05 (Ar═CHx2), 124.77 (Ar═CH), 121.92 (Ar═CH), 54.80 (–OCH_3_). HR‐MS Calcd. for: C_18_H_15_N_5_O_5_S [M + H] ^+^‐ 414.0867; found 414.0855.

*N*‐(6‐Methoxypyridazine‐3‐yl)‐4‐[(thiazol‐2‐ylmethylene)amino]benzene sulfonamide (C_15_H_13_N_5_O_3_S_2_) (**3c**): Preparative layer chromatography in chloroform. Pale yellow solid. Yield: 65%. Mp: 215°C–216°C. Rf: 0.68 (2:1 ethyl acetate/hexanes). UV‐Vis (THF, c: 2.66.10^–4 ^mol/L): λ_max_.(ɛ, L/mol. cm) = 200 (9428.57); 260 (11281.95); 312 (11548.87). FTIR (ATR, cm^–1^): ν 3055, 3024 (Ar–CH stretching, s); 2946 (Aliph –CH stretching, s); 1625 (C═N stretching, w); 1573 (C═C stretching, s); 1404 (SO_2_ asym stretching, m); 1157 (SO_2_ sym stretching, s); 740, 694 (monosubstituted aromatic ring out‐of‐plane CH bending, m); 840 (1,4‐disubstituted aromatic ring out‐of‐plane CH bending, m). ^1^H NMR (400 MHz, DMSO‐*d*_6_, ppm): *δ* 8.83 (s, 1H, N═CH), 8.14 (d, *J:* 2.9 Hz, 1H, Ar═CH), 8.06 (d, *J:* 2.5 Hz, 1H, Ar═CH), 7.89 (d, *J:* 8.5 Hz, 2H Ar═CH), 7.50 (d, *J:* 8.4 Hz, 2H, Ar═CH), 7.42 (d, *J:* 5.9 Hz, 1H, Ar═CH), 6.56 (d, *J:* 8.0 Hz, 1H, Ar═CH), 5.95 (s, 1H –NH); 3.84 (s, 3H, –OCH_3_). ^13^C NMR/APT (100 MHz, DMSO‐*d*_6_, ppm): *δ* 166.55 (Ar‐C), 156.12 (N═CH), 153.86 (Ar‐C), 153.08 (Ar‐C), 152.67 (Ar‐C), 146.44 (Ar═CH), 145.58 (Ar═CH), 138.83 (Ar‐C), 128.97 (Ar═CHx2), 128.53 (Ar.═CHx2), 125.14 (Ar═CH), 122.33 (Ar═CH), 54.81 (–OCH_3_). HR‐MS Calcd. for: C_15_H_13_N_5_O_3_S_2_ [M + H]^+^‐ 376.0533; found 376.0534.

4‐{[Benzo(*d*)thiazol‐2‐ylmethylene]amino}‐*N*‐(6‐methoxypyridazine‐3‐yl)benzene sulfonamide (C_19_H_15_N_5_O_3_S_2_) (**3d**): Recrystallization from methanol. Pale yellow solid. Yield: 59%. Mp: 227°C–230°C. Rf: 0.52 (2:1 ethyl acetate/hexanes). UV‐Vis (THF, 2.35 × 10^−4^ mol L^–1^): *λ*_max_ (ɛ, L/mol. cm) 200 (8165), 253 (10 761), 293 (12 063), 353 (974). FTIR (ATR, cm^–1^): ν 3232 (–NH stretching, m), 3078–3032 (Ar–CH stretching, m), 2931 and 2862 (Aliph –CH stretching, w), 1643 (C═N stretching, w), 1589 and 1460 (Ar C═C stretching, m), 1396 (–SO_2_ asym stretching, s), 1296 (C–N stretching, s), 1134 (–SO_2_ sym stretching, s), 1087 (C–O sym stretching, s), 1010 (1,4‐disubstituted aromatic ring in‐plane CH bending, m), 817 and 732 (1,4‐disubstituted aromatic ring out‐of‐plane CH bending, s). ^1^H NMR (400 MHz, DMSO‐*d*_6_, ppm): *δ* 8.98 (s, 1H, N═CH), 8.15 (d, *J*: 8.7 Hz, 1H, Ar═CH), 8.01 (d, *J*: 8.2 Hz, 1H, Ar═CH), 7.77 (s, 1H, NH), 7.60 (d, *J*: 8.4 Hz, 2H, Ar═CH), 7.56–7.48 (m, 2H, Ar═CH), 7.29 (d, 2H, *J*: 8.8 Hz, Ar═CH), 6.93 (d, *J*: 8.8 Hz, 1H, Ar═CH), 6.17 (d, *J*: 8.1 Hz, 1H, Ar═CH), 3.85 (s, 3H, OCH_3_). ^13^C NMR/APT (100 MHz, DMSO‐*d*_6_, ppm): *δ* 167.03 (N═CH), 159.97 (N═C–S), 155.80 (Ar‐C), 153.86 (Ar‐C), 152.20 (Ar‐C), 149.26 (Ar‐C), 138.31 (Ar‐C), 135.24 (Ar‐C), 128.15 (Ar═CHx2), 127.95 (Ar═CH), 127.54 (Ar═CHx2), 125.82 (Ar═CHx2), 124.64 (Ar═CH), 123.34 (Ar═CH), 122.59 (Ar═CH), 55.04 (–OCH_3_). HR‐MS Calcd. for C_19_H_15_N_5_O_3_S_2_ [M + H]^+^‐ 426.0689; found 426.0673.

### 4.2 Biological Activity

#### 4.2.1 Cell Culture and Cytotoxic Activity (MTT and WST‐8 Assay)

Colon cancer cell lines DLD‐1 (ATCC:CCL‐221), HT‐29 (ATTC:HTB‐38), and normal cell line CCD‐18Co (ATCC:CRL‐1459) used in this study were obtained from Gebze Technical University and SAP Institute (Türkiye). Cell lines were grown using RPMI 1650 and Dulbecco's Modified Eagle Medium (DMEM) media containing 0.2 g/100 mL sodium bicarbonate, 10% FBS (Fetal bovine serum) and 1% penicillin/streptomycin in 25 or 75 flasks in an incubator with 5% CO_2_ and 37°C. In the study, different doses of the compounds, 1.56 μM, 3.12 μM, 6.25 μM, 12.5 μM, 25 μM, 50 μM, 100 μM for the MTT assay and 6.25 μM, 12.5 μM, 25 μM, 50 μM, 100 μM for the WST‐8 assay (triplicate: technical replicates), and 0.1% DMSO (Dimethyl Sulfoxide) control were used. Only cells and medium were used as negative control. Cells were seeded at 5.000 cells per well, and MTT (Thiazolyl blue tetrazolium bromide) [77] and WST‐8 (Water‐soluble tetrazolium‐8) [78] tests were applied for cytotoxic activity determinations. Absorbance was measured at 570 nm for the MTT test (Sigma‐Aldrich) and 450 nm for the WST‐8 test (Sigma‐Aldrich), and the ANOVA analysis, %viability, LogIC_50_ and IC_50_ values of the compounds (**1**: starting, **3a–d**: target) were calculated with the Prism 9 program (Figures 1, 2, 3, Supporting Information S1: Table S1–S5).

#### 4.2.2 RNA Isolation, cDNA Synthesis, and qRT‐PCR Analysis of Antioxidant and Apoptotic Genes

The most effective doses of the compounds (in DLD‐1 cell; 12.5 μM for compound **1**, 12.5 μM for compound **3a**, 12.5 μM for compound **3b**, 25 μM for compound **3c**, 100 μM for compound **3d**, and in the HT‐29 cell; 12.5 μM for compound **1**, 12.5 μM for compound **3a**, 25 μM for compound **3b**, 25 μM for compound **3c**, and 100 μM for compound **3d**) were selected according to the cytotoxic activity results by MTT and WST‐8 assay. DLD‐1 and HT‐29 (colon adenocarcinoma) cells were incubated at 37°C for 24 h and RNA isolation (Thermo Fisher) was performed by seeding 2 × 10^6^ cells per well into 6‐well plates [79].

cDNA synthesis (Thermo Fisher) was performed from total RNAs isolated from DLD‐1 and HT‐29 cells, and Real Time PCR (Roche Lightcycler 96) analyses were performed with the Thermo Fisher kit protocol. The sequences of the genes were determined through the NCBI database (https://www.ncbi.nlm.nih.gov/tools/primer-blast). Genes, sequences, accessions, and length of primers are presented in Supporting Information S1: Table S7.

The mRNA expression levels (single replicate) of the apoptotic genes (BAX, BCL‐2, p53, Caspase‐3, Caspase‐8, Caspase‐9) and antioxidant genes (SOD‐1, SOD‐2, CAT, GSS) of the compounds were investigated by the real‐time PCR (qPCR) method, and heatmap maps were created (Tables 1 and 2). β‐Actin (ACTB) was used as a housekeeping control gene. Calculation of 2^–ΔΔCT^ value to determine gene expression was performed by the method of Livak and Schmittgen, 2001 [80].

#### 4.2.3 Protein Isolation, ELISA Analysis of Antioxidant and HDAC Activities, Western Blot Analysis of Apoptotic Activities

The most effective doses of the compounds were selected based on cytotoxic activity results. DLD‐1 and HT‐29 (colon adenocarcinoma) cells were incubated at 37°C for 24 h, and protein isolation (Thermo Fisher) was performed with RIPA buffer by seeding 6 × 10^6^ cells in 75 flasks [79]. The isolated proteins (single replicate) were used for ELISA analysis for their total antioxidant and HDAC activities and for western blot analysis for their apoptotic activities (P38/MAPK and ERK1).

The kit protocol was applied for total antioxidant activity (Rel Assay) by ELISA analysis (double replicate) in DLD‐1 and HT‐29 cells. 18 μL of protein for each compound, ddH_2_O for blank, and the standard were added to the 96‐well plate. Then, 300 μL Reagent 1 was added to the wells and mixed. The plate was incubated for 30 s, and absorbance was measured at 600 nm (A1). A total of 45 μL of Reagent 2 was added to the wells and mixed, and the plate was incubated at 37°C for 5 min. The absorbance of the plate was measured again at 600 nm (A2). Total Antioxidant activity in DLD‐1 and HT‐29 cells was determined with the ChroMate 4300 device by calculation on Excel (Figure 4a,b). Calculation: A2‐A1: ΔAbs of standard or sample or H_2_O; Results: [ΔAbs H_2_O – ΔAbs Sample]/[ΔAbs H_2_O – ΔAbs Standard].

The kit protocol was applied for HDAC activity (BioVision) by ELISA analysis (double replicate) in DLD‐1 and HT‐29 cells. A volume of 10 μL of HeLa nuclear extract was used as a positive control. Trichostatin A and the protein obtained with cells and medium only were used as negative controls. A total of 85 μL of protein for each compound and 10 μL of HDAC assay buffer were added to the 96‐well plate. Then, 5 μL of HDAC substrate was added to the wells and pipetted, and the plate was incubated at 37°C for 1 h. A volume of 10 μL of Lysine developer was added to the wells and pipetted and incubated again at 37°C for 30 min. HDAC activity in DLD‐1 and HT‐29 cells was determined with the ChroMate 4300 device by absorbance measurement at 405 nm (Figure 5a,b).

After the separation gel (10%) and loading gel (4%) were prepared, they were placed in the vertical electrophoresis tank. Proteins of each compound were mixed with sample loading buffer (200 μL 50% Glycerol, 100 μL 1 M Tris, 100 μL 10% SDS, 50 μL 0.1% Bromphenol, and 50 μL DTT) in a ratio of 30:30. After the samples were kept in a 95°C dry block heater for 5 min, 40 μL was loaded into each well. Protein bands were transferred to the membrane by applying 120 V current for 3.5 h and using the Bio‐Rad Transblot Turbo Transfer system. The membrane was washed with 30 μL TBST for 10 min and treated with blocking buffer (50 mL TBST + 2.5 g milk powder) for 1 h. The membrane was washed with TBST and added antibody‐buffer (20 mL 1 X TBST, 0.2 gr milk powder), 20 μL P38/MAPK, ERK1 (RD, anti ‐h/mPhospoho‐ERK1), and anti‐ß‐actin antibody and left to incubate for 1 night. After incubation, the membrane was washed again with TBST. 20 mL 1 X TBST, 0.2 gr milk powder, 4 μL secondary antibody were added to the membrane and waited for 1 h. After 1 h, it was washed again with TBST. Peroxide buffer and Luminol/Enhancer (Thermo) were added to the membrane, and imaging was performed on the chemiluminescence device (Vilber Lourmat/FX5) [81, 82]. Calculation of protein expression levels in membrane images was carried out using Image J and Excel programs (Figure 6a,b).

## Conflicts of Interest

The authors declare no conflicts of interest.

## Supporting information


Supporting File 1

Supporting File 2
