# Supplementary material for: Synthesis and Characterization of Sulfonamide‐Schiff Bases, and Investigation of Cytotoxic, Antioxidant, HDAC, and Apoptotic Activities in Human Colon Cancer Cells (DLD‐1 and HT‐29)
Source: Arch Pharm (Weinheim). 2026 Apr 10;359(4):e70235. doi: 10.1002/ardp.70235 (PMC13068298; doi:10.1002/ardp.70235)
Supplement: Supplementary file 1 — Supporting File 1 [file ARDP-359-e70235-s002.docx]

23SSSS1S

**SUPPLEMENTARY FILE**

**Synthesis and characterization of sulfonamide-Schiff bases, and investigation of cytotoxic, antioxidant, HDAC and apoptotic activities in human colon cancer cells (DLD-1 and HT-29)**

Seda Mesci^1a^*, Berna Kocaman^1b^, Aliye Gediz Erturk^c^*, Emine Bagdatli^c^, Burak Yazgan^d^, Tuba Yildirim^b,e^

^a^ Project Coordination and Guidance Office, Rectorate, Hitit University, 19100, Çorum, Turkey

^b^ Department of Biotechnology, Institute of Science, Amasya University, 05100 Amasya, Turkey

^c^ Department of Chemistry, Faculty of Science and Arts, Ordu University, 52200, Ordu, Turkey

^d^ Department of Medical Services and Techniques, Sabuncuoğlu Serefeddin Health Services Vocational School, Amasya University, 05100, Amasya, Turkey

^b,e^ Department of Biology, Faculty of Art and Science, Amasya University, 05100 Amasya, Turkey

| Seda Mesci | : [sedamesci@hitit.edu.tr](mailto:sedamesci@hitit.edu.tr), 0000-0002-5440-302X |
| --- | --- |
| Berna Kocaman | : [bernakocaman111@gmail.com](mailto:bernakocaman111@gmail.com), 0000-0002-5578-8697 |
| Aliye Gediz Erturk | : [aliyerturk@odu.edu.tr](mailto:aliyerturk@odu.edu.tr), 0000-0003-0831-7056 |
| Emine Bagdatli | : ebagdatli@odu.edu.tr, 0000-0001-5946-2988 |
| Burak Yazgan | : [burak.yazgan@amasya.edu.tr](mailto:burak.yazgan@amasya.edu.tr), 0000-0003-0717-7768 |
| Tuba Yildirim | : [tuba.yildirim@amasya.edu.tr](mailto:tuba.yildirim@amasya.edu.tr), 0000-0001-8575-4802 |

^1^ Both authors are co-first authors.

^a^* Corresponding author (Biology)

Phone: +90 364 2192892 e-mail: [sedamesci@hitit.edu.tr](mailto:sedamesci@hitit.edu.tr)

^c^* Corresponding author (Chemistry)

Phone: +90 262 6053011 e-mail: [aliyerturk@odu.edu.tr](mailto:aliyerturk@odu.edu.tr)

**CONTENTS**

[**Figure S1:** Scheme of compounds **3a-3d** synthesis……………………………………..........](#_Toc151936716) .2

[**Figure S2:** ^1^H NMR spectrum of compound **3a**](#_Toc151936716) in DMSO-*d_6_* 3

[**Figure S3:** APT-^13b^ NMR spectrum of compound **3a**](#_Toc151936716) in DMSO-*d_6_* 3

[**Figure S4:** ^1^H NMR spectrum of compound **3b**](#_Toc151936716) in DMSO-*d_6_* 4

[**Figure S5:** APT-^13b^ NMR spectrum of compound **3b**](#_Toc151936716) in DMSO-*d_6_* 4

[**Figure S6:** ^1^H NMR spectrum of compound **3c**](#_Toc151936716) in DMSO-*d_6_* 5

[**Figure S7:** APT-^13b^ NMR spectrum of compound **3c**](#_Toc151936716) in DMSO-*d_6_* 5

[**Figure S8:** ^1^H NMR spectrum of compound **3d**](#_Toc151936716) in DMSO-*d_6_* 6

[**Figure S9:** APT-^13b^ NMR spectrum of compound **3d**](#_Toc151936716) in DMSO-*d_6_* 6

**Figure S10:** LC-MS/TOF Total Ion Chromatogram (TIC) – Purity Profile (Rt: 0.16 min.) and LC-MS/TOF chromatogram of compound **3a** 7

**Figure S11:** LC-MS/TOF Total Ion Chromatogram (TIC) – Purity Profile (Rt: 0.11 min.) and LC-MS/TOF chromatogram of compound **3b** 8

**Figure S12:** LC-MS/TOF Total Ion Chromatogram (TIC) – Purity Profile (Rt: 0.13 min.) and LC-MS/TOF chromatogram of compound **3c** 9

**Figure S13:** LC-MS/TOF Total Ion Chromatogram (TIC) – Purity Profile (Rt: 0.12 min.) and LC-MS/TOF chromatogram of compound **3d** 10

[**Figure S14:** FT-IR spectrum of compound **1**](#_Toc151936716)  11

**Figure S15:** FT-IR spectrum of compound **3a** 11

**Figure S16:** FT-IR spectrum of compound **3b** 12

**Figure S17:** FT-IR spectrum of compound **3c** 12

**Figure S18:** FT-IR spectrum of compound **3d** 13

**Table S1:** Cytotoxic activities and IC50 values of compounds with %viability rates at different doses in colon cancer (DLD-1) cells by MTT assay 14

**Table S2:** Cytotoxic activities and IC50 values of compounds with %viability rates at different doses in colon cancer (HT-29) cells by MTT assay 14

**Table S3:** Cytotoxic activities of compounds with %viability rates at different doses in normal colon (CCD-18Co) cells by MTT assay 15

**Table S4:** Cytotoxic activities and IC50 values of compounds with %viability rates at different doses in colon cancer (DLD-1) cells by WST-8 assay 15

**Table S5:** Cytotoxic activities and IC50 values of compounds with %viability rates at different doses in colon cancer (HT-29) cells by WST-8 assay 16

**Table S6.** Selectivity index (SI) values of compounds in colon cancer cells (DLD-1 and HT-29) compared to normal colon cells (CCD-18Co) by MTT assay……………………………..16

**Table S7:** Primer sequences, accessions, lengths and melting temperature (^o^C) of the genes used in qRT-PCR analysis in colon cancer cells 17

**Figure S1.** Scheme of compounds **3a-3d** synthesis


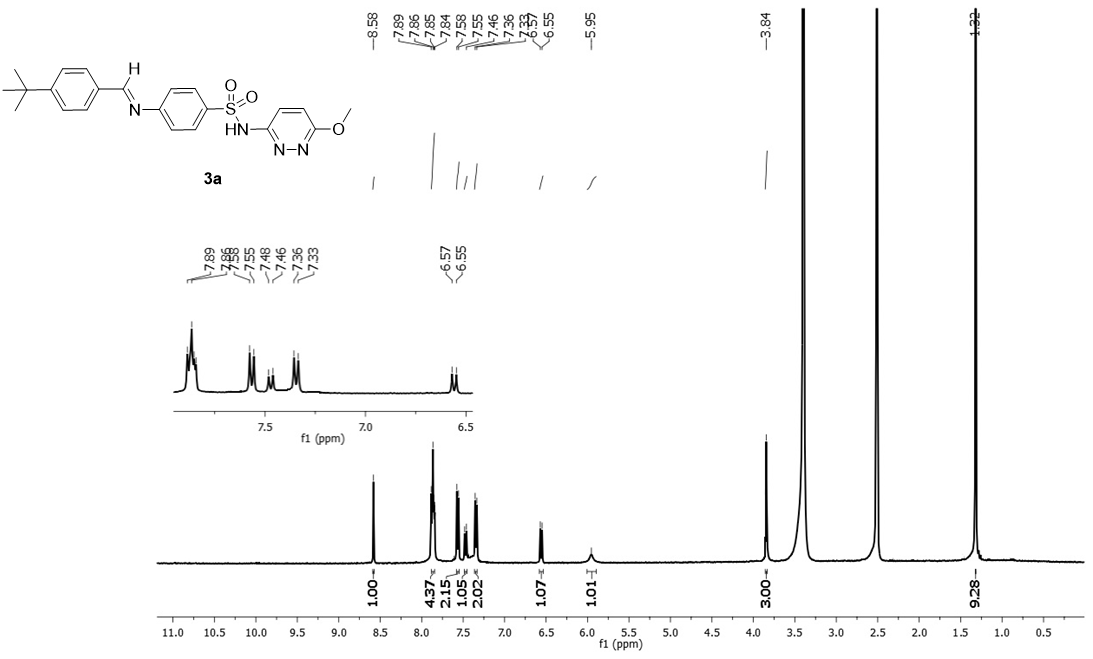


**Figure S2.** ^1^H NMR spectrum of compound **3a** in DMSO-*d_6_*


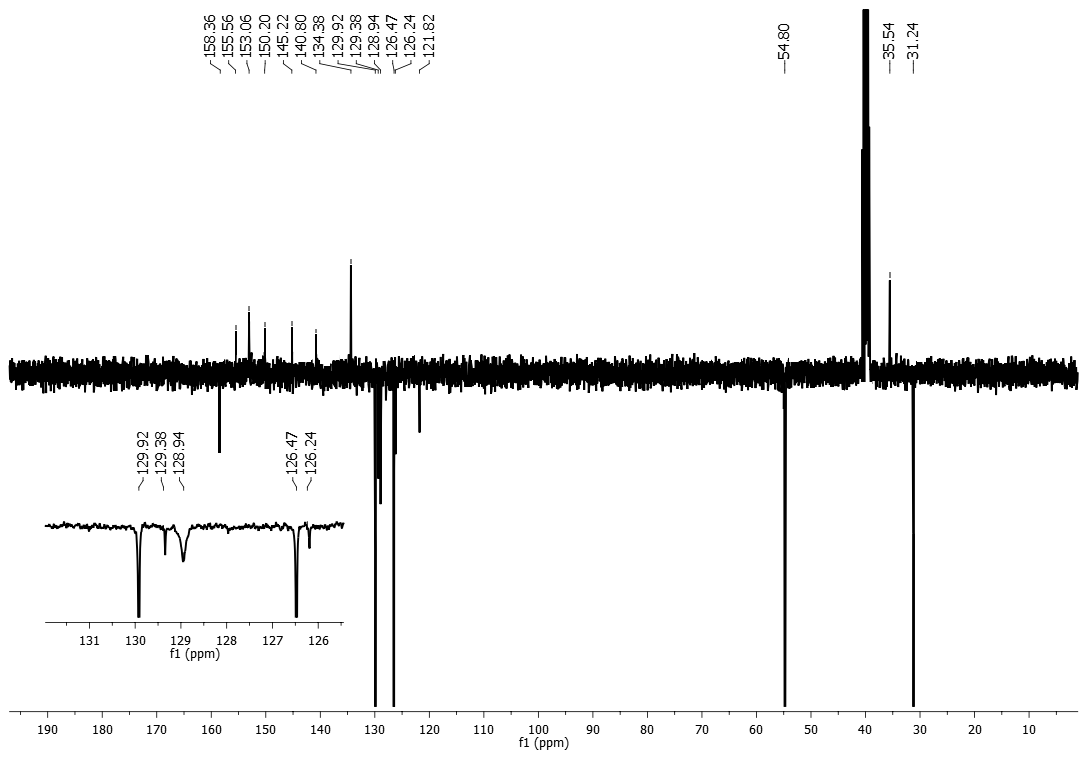


**Figure S3.** APT-^13^C spectrum of compound **3a** in DMSO-*d_6_*


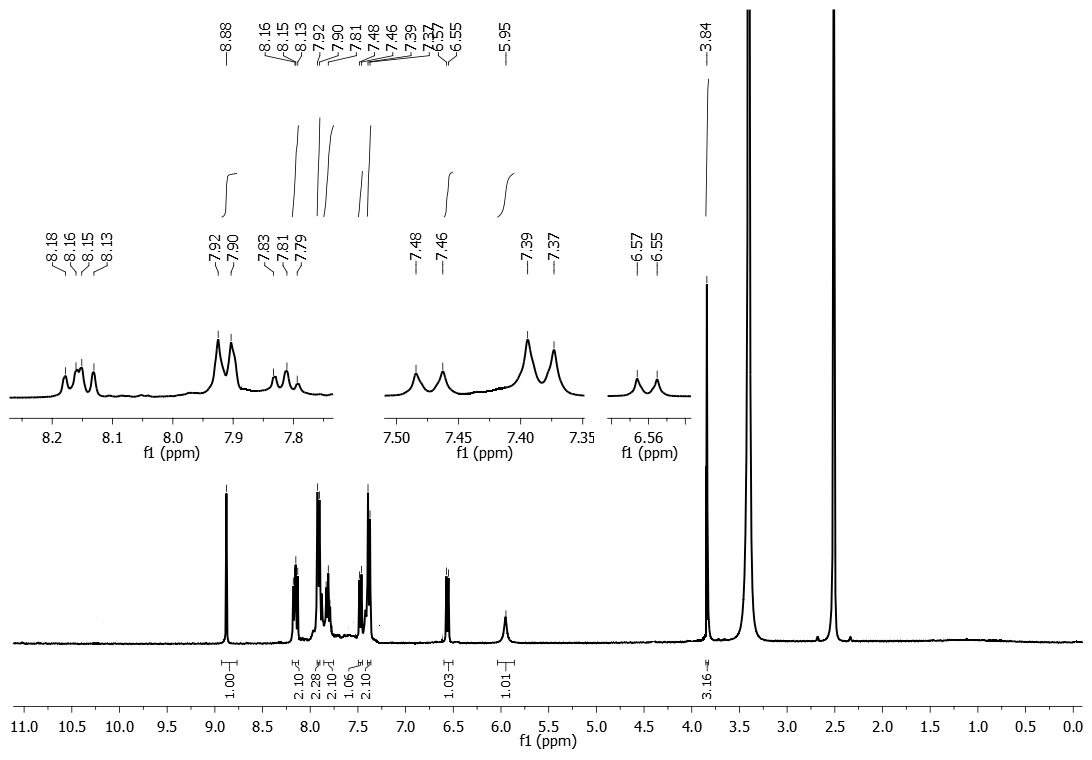


**Figure S4.** ^1^H NMR spectrum of compound **3b** in DMSO-*d_6_*


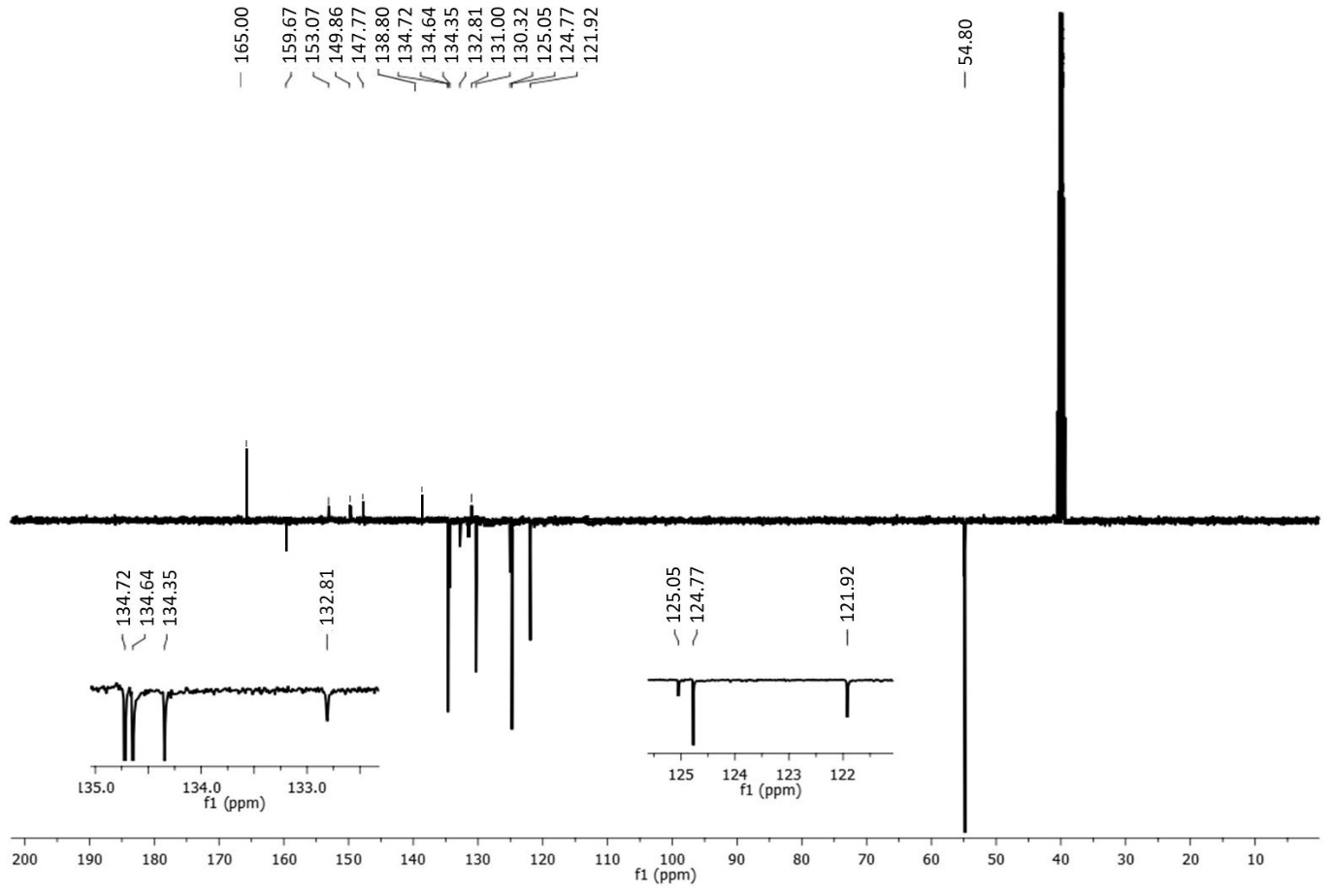


**Figure S5.** APT-^13^C spectrum of compound **3b** in DMSO-*d_6_*


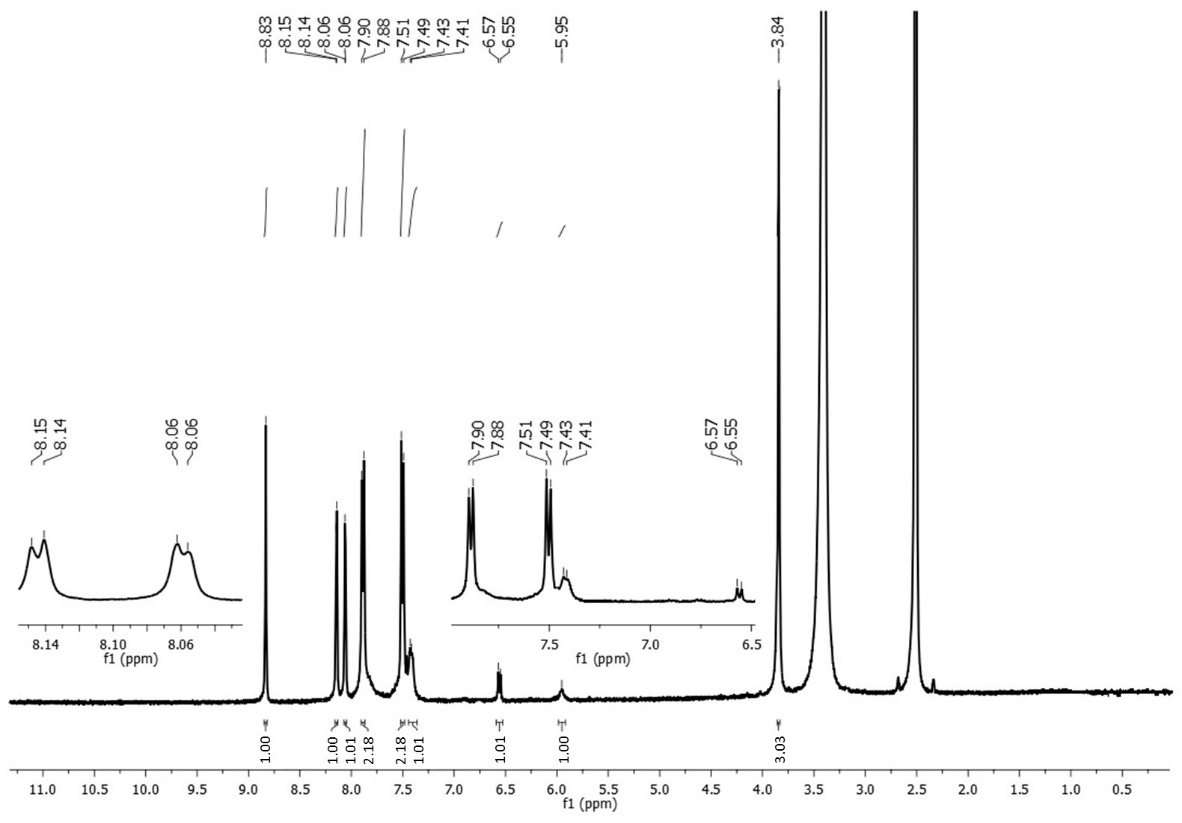


**Figure S6.** ^1^H NMR spectrum of compound **3c** in DMSO-*d_6_*


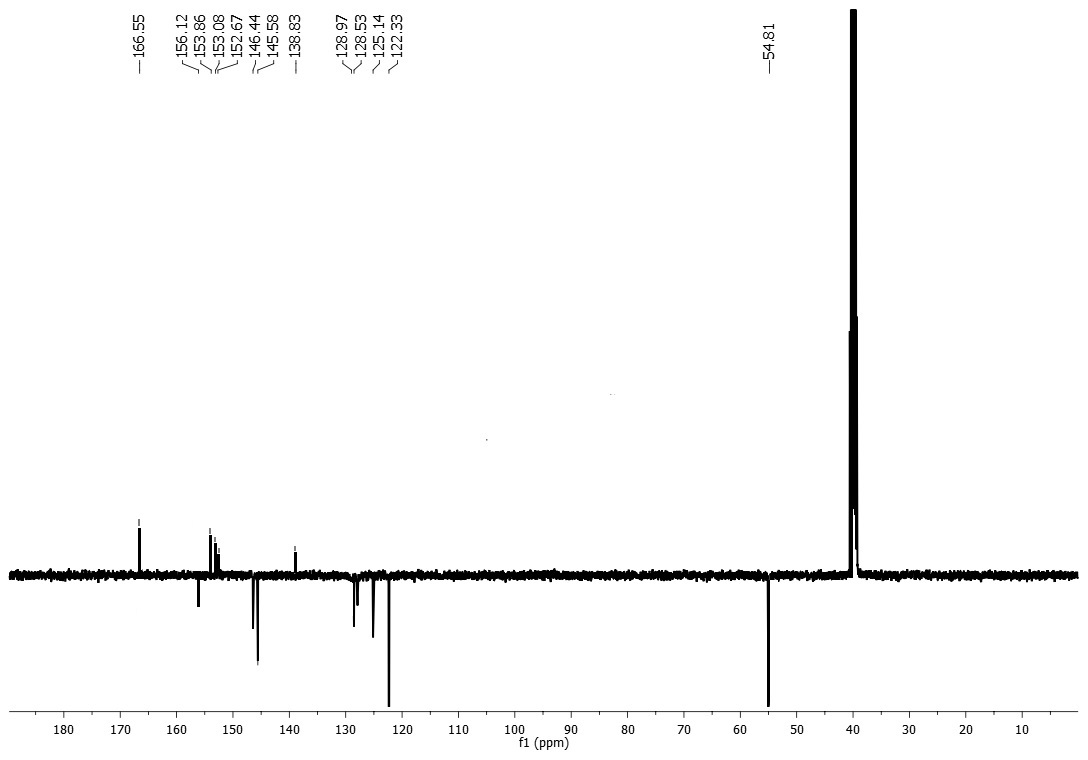


**Figure S7.** APT-^13^C spectrum of compound **3c** in DMSO-*d_6_*


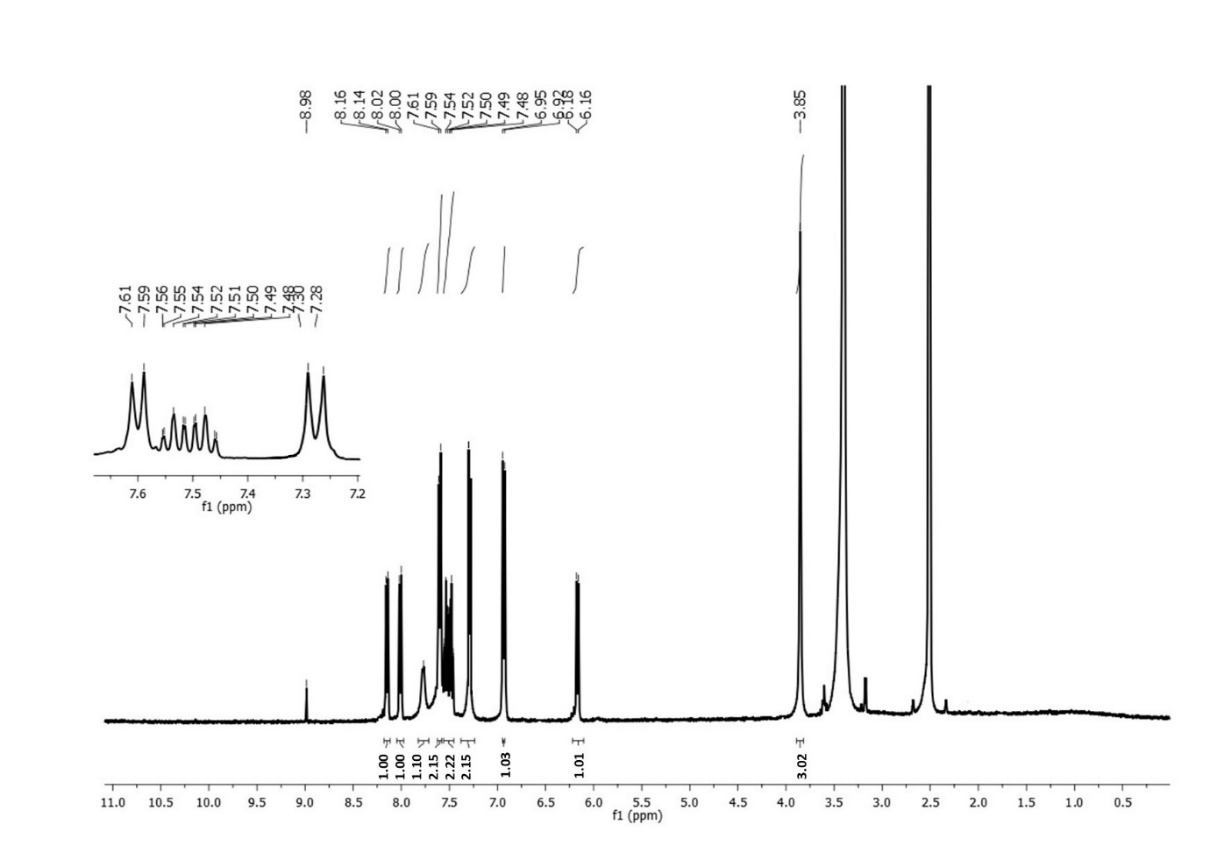


**Figure S8.** ^1^H NMR spectrum of compound **3d** in DMSO-*d_6_*


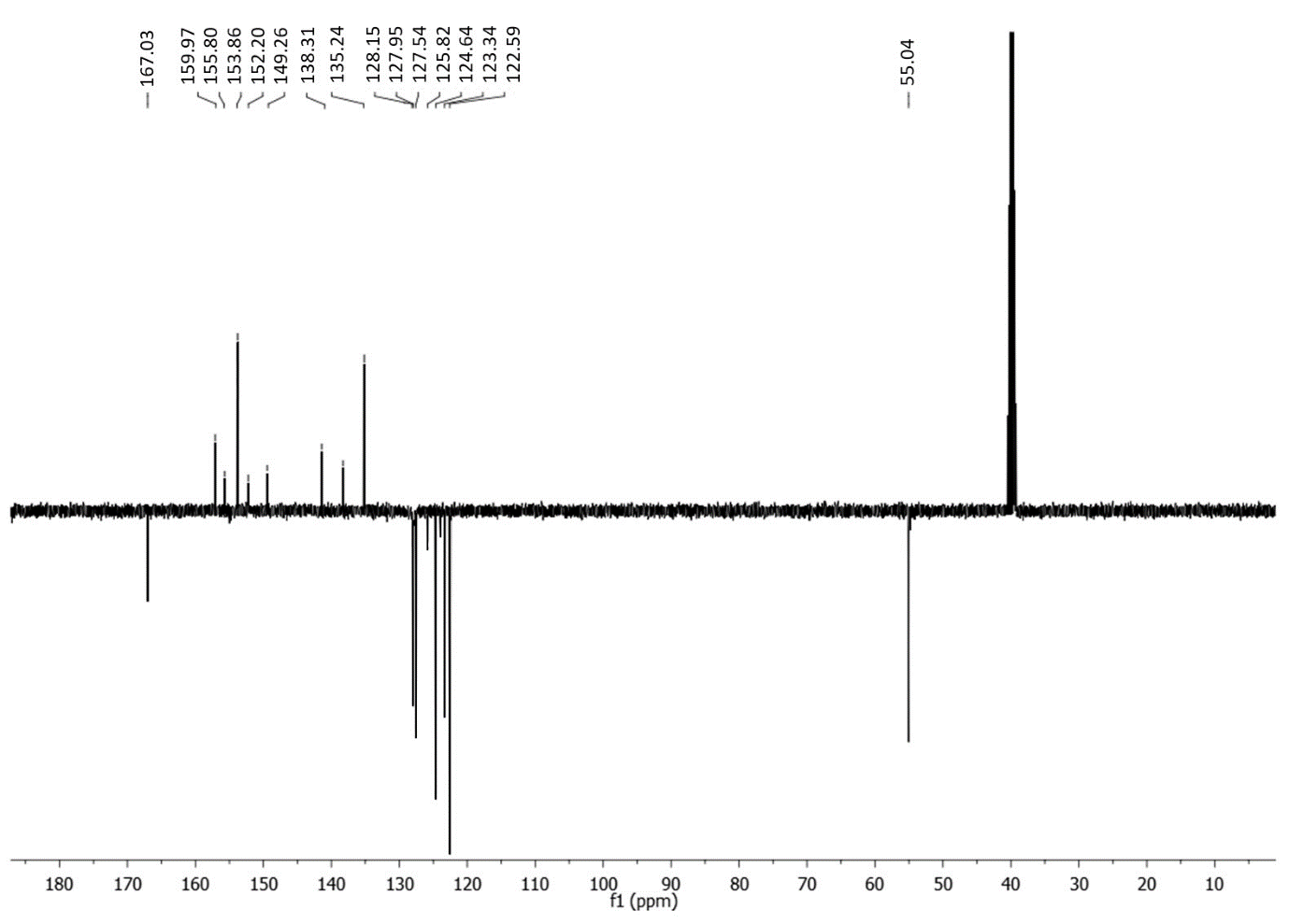


**Figure S9.** APT-^13^C spectrum of compound **3d** in DMSO-*d_6_*

**
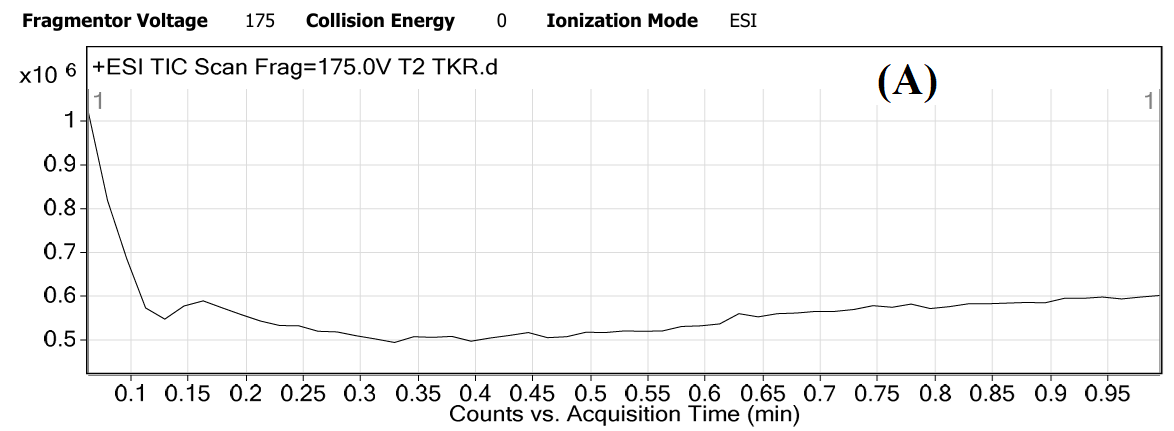
**

**
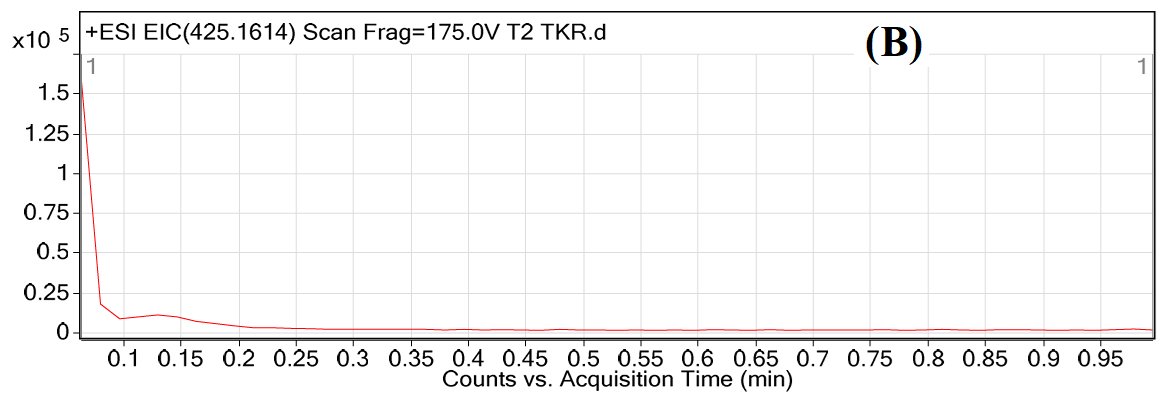
**


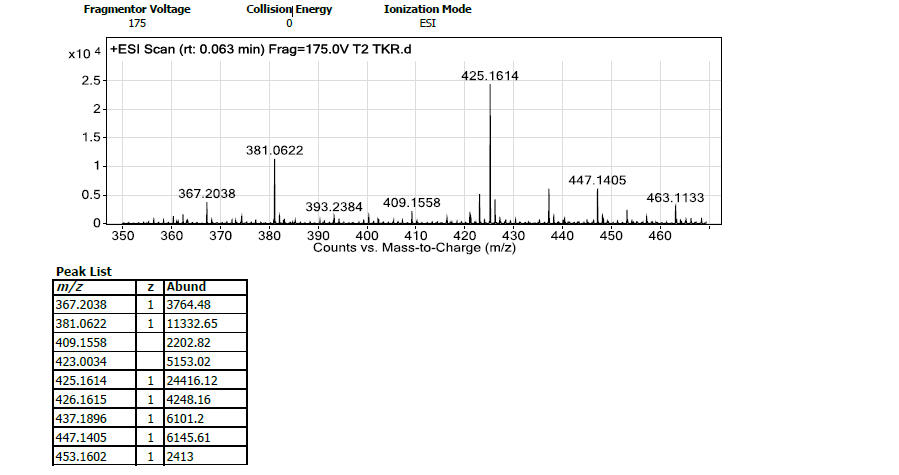

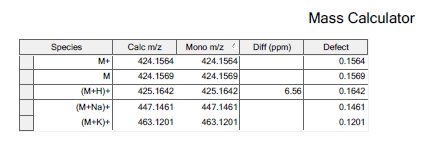

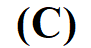


**Figure S10:** LC-MS/TOF Total Ion Chromatogram (TIC) – Purity Profile (Rt: 0.16 min.) and LC-MS/TOF chromatogram of compound **3a**

**(A)** LC-MS/TOF Total Ion Chromatogram (TIC)

**(B)** Extracted ion chromatogram (EIC) corresponding to the protonated molecular ion [m+H]+ (m/z = 425.1614), confirming the presence of a single dominant peak for the target compound.

**(C)** Purity Profile (Rt: 0.16 min.) and LC-MS/TOF chromatogram of compound **3a,** respectively


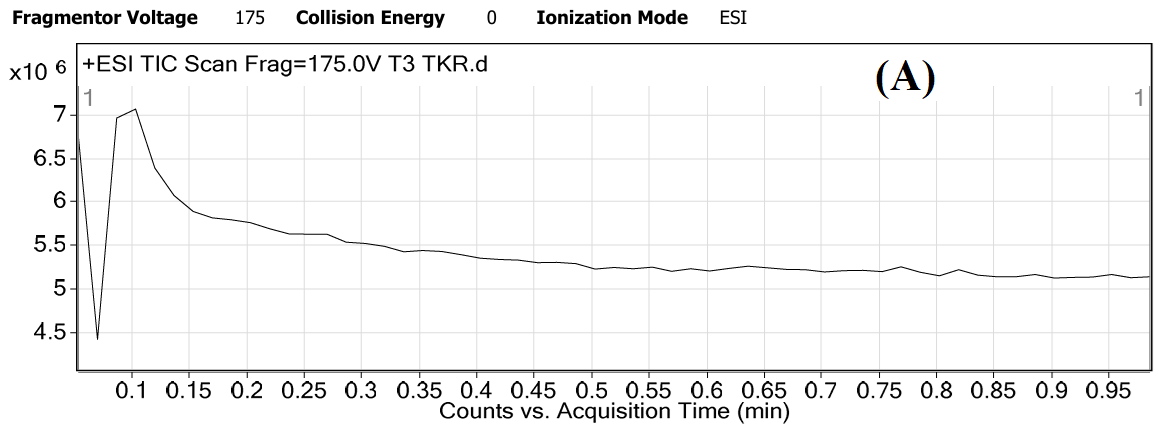


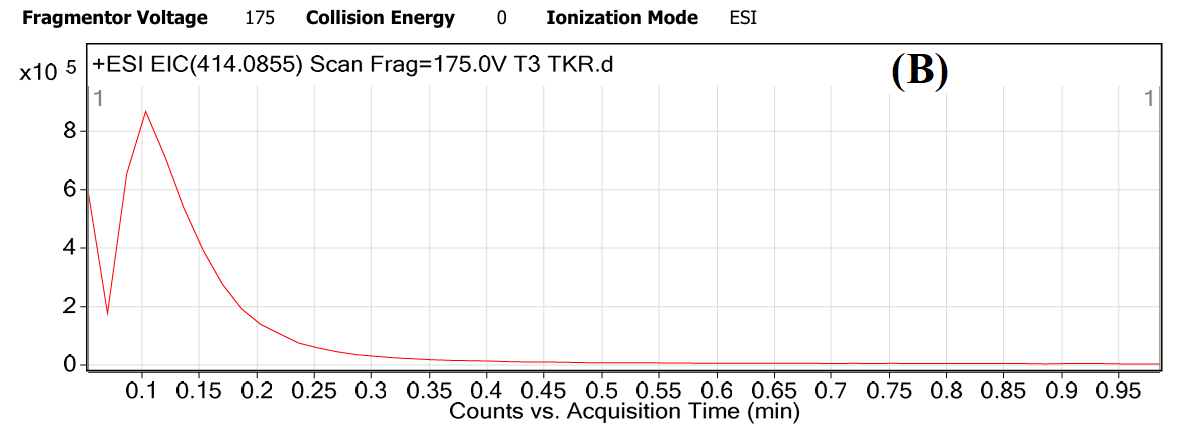


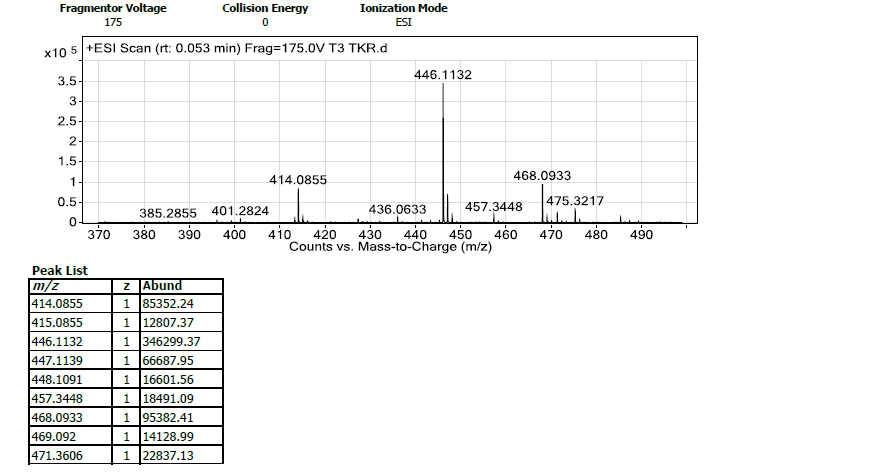

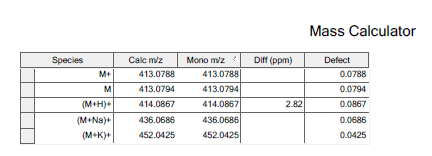

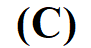


**Figure S11:** LC-MS/TOF Total Ion Chromatogram (TIC) – Purity Profile (Rt: 0.11 min.) and LC-MS/TOF chromatogram of compound **3b**

**(A)** LC-MS/TOF Total Ion Chromatogram (TIC)

**(B)** Extracted ion chromatogram (EIC) corresponding to the protonated molecular ion [m+H]+ (m/z = 414.0855), confirming the presence of a single dominant peak for the target compound.

**(C)** Purity Profile (Rt: 0.11 min.) and LC-MS/TOF chromatogram of compound **3b,** respectively


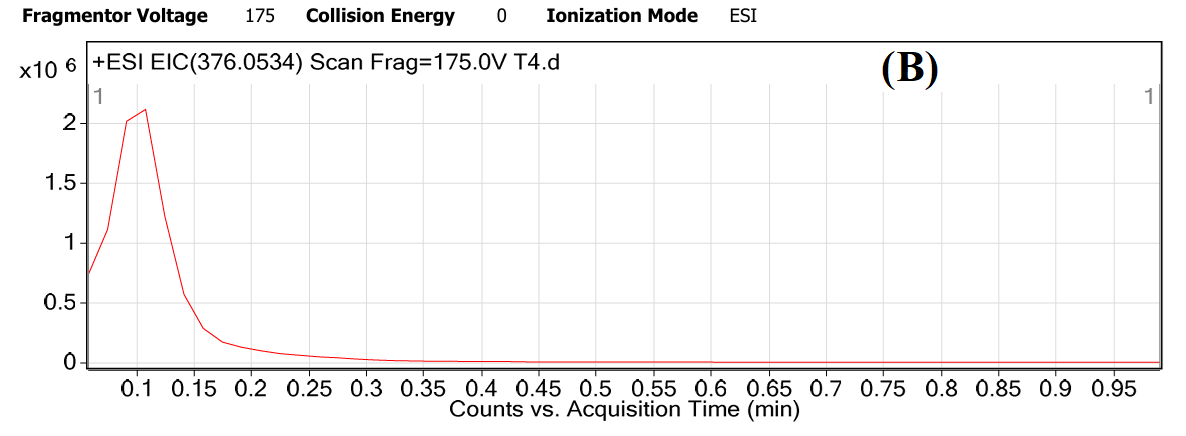

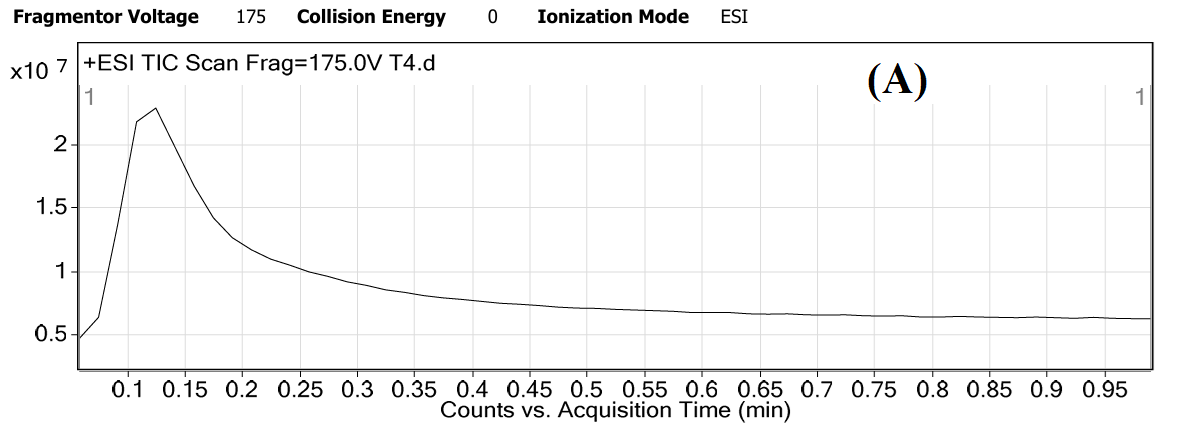


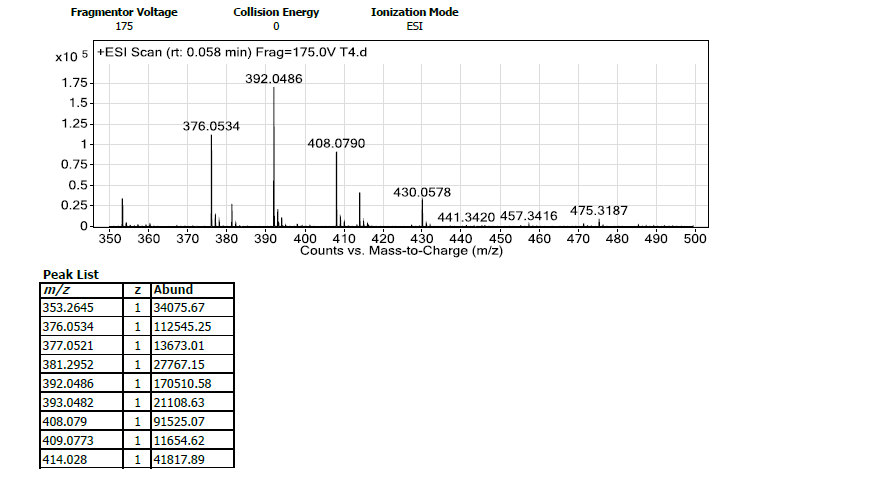

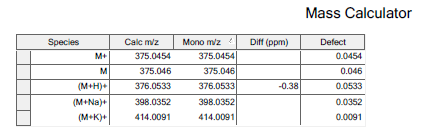

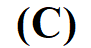


**Figure S12:** LC-MS/TOF Total Ion Chromatogram (TIC) – Purity Profile (Rt: 0.13 min.) and LC-MS/TOF chromatogram of compound **3c**

**(A)** Total Ion Chromatogram (TIC)

**(B)** Extracted ion chromatogram (EIC) corresponding to the protonated molecular ion [m+H]+ (m/z = 376.0534), confirming the presence of a single dominant peak for the target compound

**(C)** Purity Profile (Rt: 0.13 min.) and LC-MS/TOF chromatogram of compound **3c,** respectively


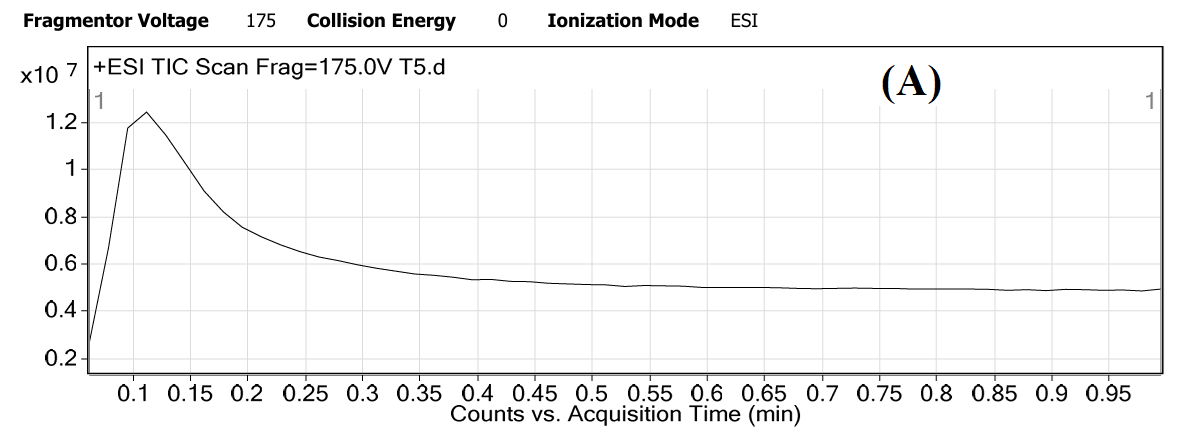


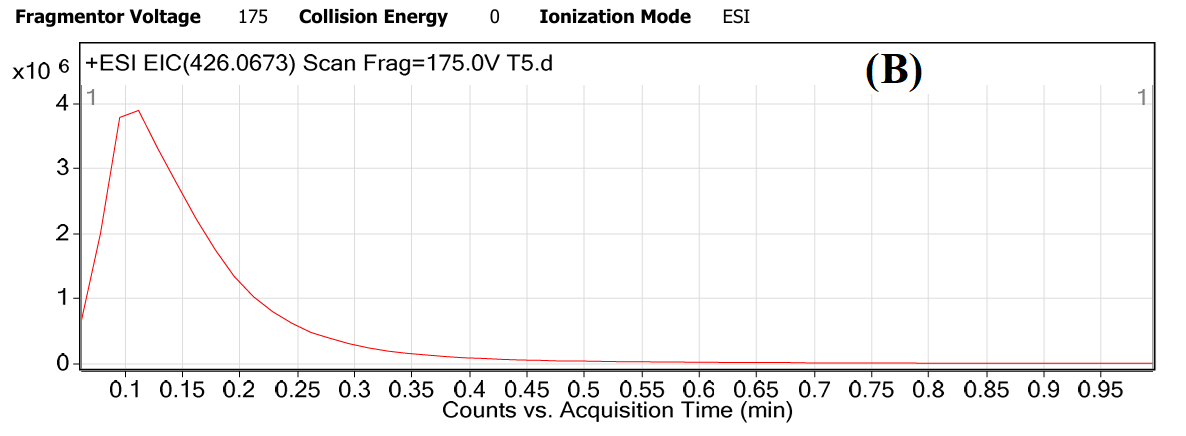


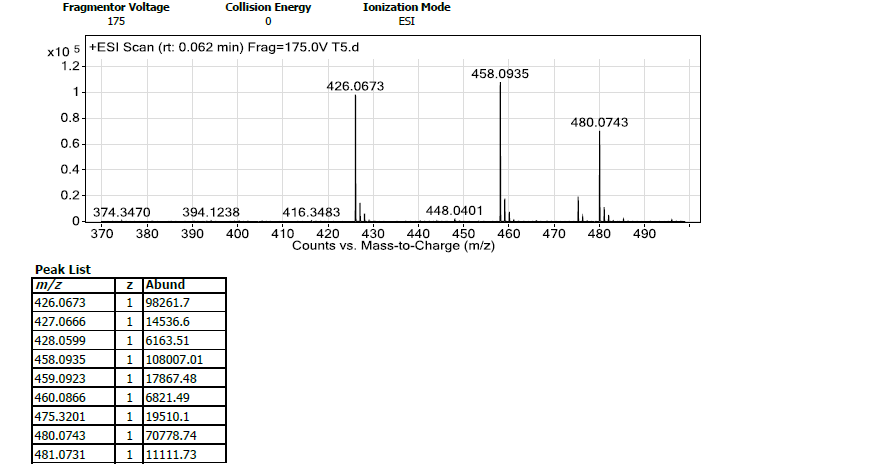

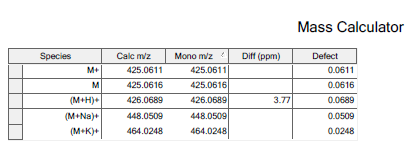

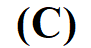


**Figure S13:** LC-MS/TOF Total Ion Chromatogram (TIC) – Purity Profile (Rt: 0.12 min.) and LC-MS/TOF chromatogram of compound **3d**

**(A)** Total Ion Chromatogram (TIC)

**(B)** Extracted ion chromatogram (EIC) corresponding to the protonated molecular ion [m+H]+ (m/z = 426.0673), confirming the presence of a single dominant peak for the target compound.

**(C)** Purity Profile (Rt: 0.12 min.) and LC-MS/TOF chromatogram of compound **3d,** respectively


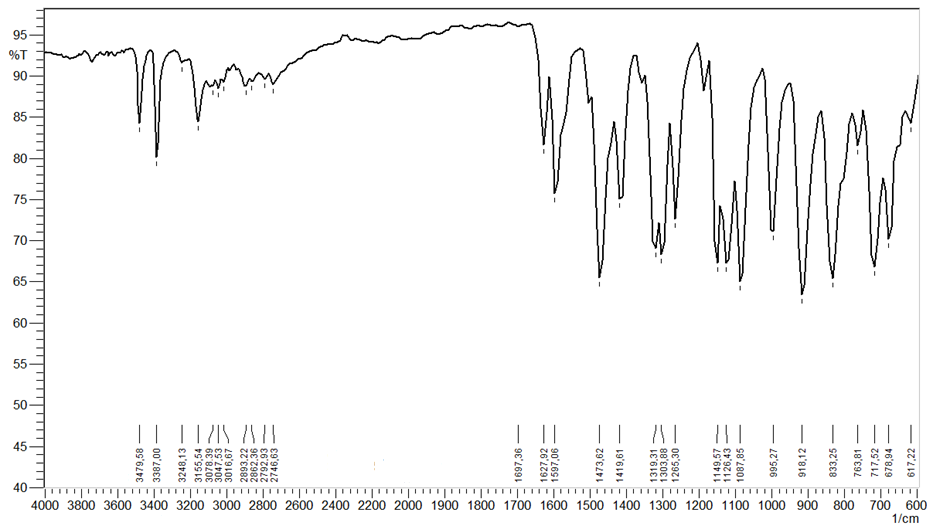


**Figure S14.** FT-IR spectrum of compound **1**


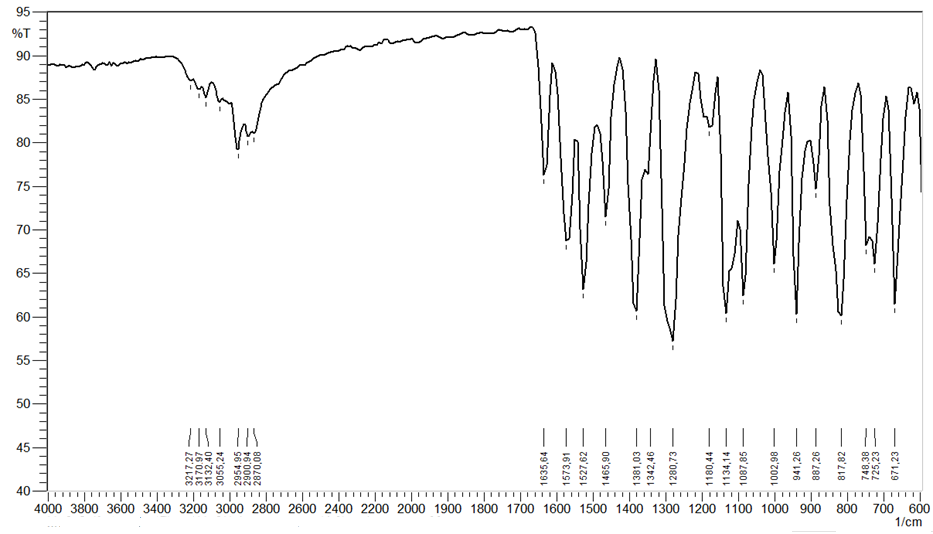


**Figure S15.** FT-IR spectrum of compound **3a**

**Figure S16.** FT-IR spectrum of compound **3b**


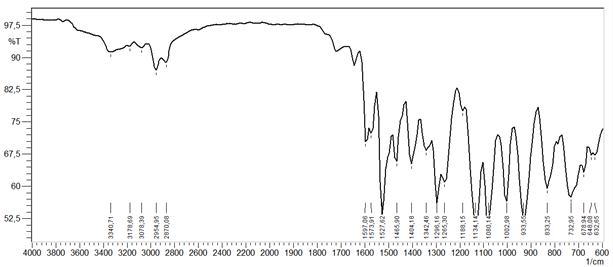

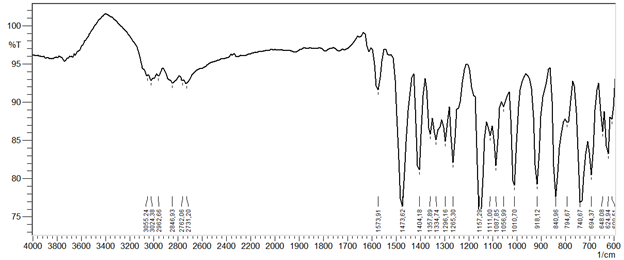


**Figure S7.** FT-IR spectrum of compound **3c**

**Figure S18.** FT-IR spectrum of compound **3d**


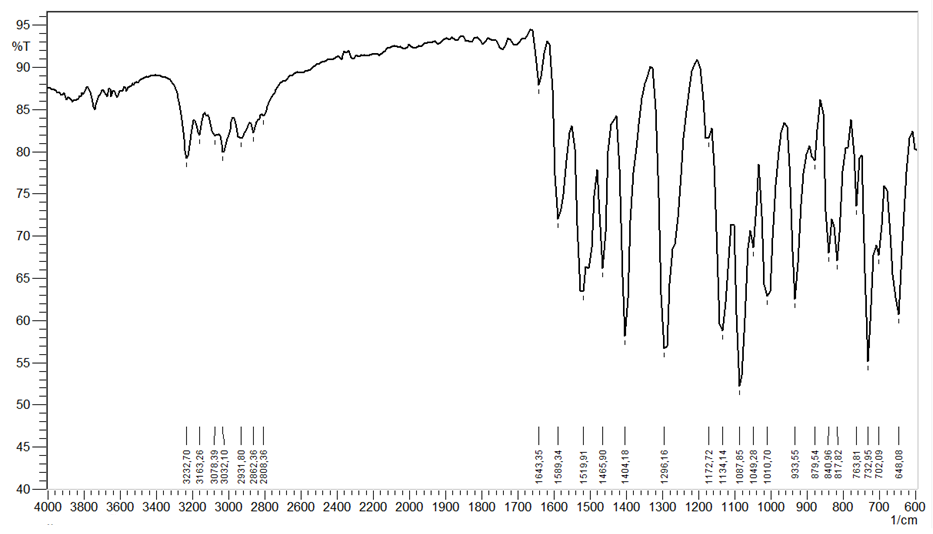


**Table S1.** Cytotoxic activities and IC_50_ values of compounds with %viability rates at different doses in colon cancer (DLD-1) cells by MTT assay

| **DLD-1 cell** | **Compound 1** | **Compound 3a** | **Compound 3b** | **Compound 3c** | **Compound 3d** |
| --- | --- | --- | --- | --- | --- |
| **^[a]^ (-) Control** | 100 | 100 | 100 | 100 | 100 |
| **^[b]^ DMSO Control** | 88.53 | 80.85 | 85.33 | 77.59 | 77.41 |
| **1.56µM** | 84.67 | 95.65 | 70.42 | 53.35 | 92.29 |
| **3.12µM** | 79.73 | 91.87 | 68.96 | 44.33 | 88.33 |
| **6.25µM** | 64.10 | 89.67 | 62.39 | 42.43 | 88.20 |
| **12.5µM** | 59.76 | 85.18 | 62.24 | 41.37 | 87.35 |
| **25µM** | 47.73 | 74.28 | 58.15 | 39.90 | 83.45 |
| **50µM** | 46.98 | 72.65 | 49.57 | 38.61 | 73.84 |
| **100µM** | 39.51 | 67.53 | 48.25 | 37.70 | 69.45 |
| **^[c]^ IC50 value** | 9.63 | >100 | 15.54 | 3.94 | >100 |

^[a]^ The result are expressed as (-) Control=Cell and medium ^[b]^ DMSO Control =0.1% DMSO

^[c]^ IC_50_: Half maximal inhibitory concentration (μg/ml)

**Table S2.** Cytotoxic activities and IC_50_ values of compounds with %viability rates at different doses in colon cancer (HT-29) cells by MTT assay

| **HT29 cell** | **Compound 1** | **Compound 3a** | **Compound 3b** | **Compound 3c** | **Compound 3d** |
| --- | --- | --- | --- | --- | --- |
| **^[a]^ (-) Control** | 100 | 100 | 100 | 100 | 100 |
| **^[b]^ DMSO Control** | 81.58 | 94.25 | 90.09 | 81.44 | 74.89 |
| **1.56µM** | 77.00 | 70.42 | 83.01 | 72.44 | 71.80 |
| **3.12µM** | 66.26 | 68.96 | 61.73 | 61.14 | 69.19 |
| **6.25µM** | 62.96 | 62.39 | 53.85 | 60.38 | 68.99 |
| **12.5µM** | 58.85 | 62.24 | 52.90 | 56.79 | 66.94 |
| **25µM** | 54.56 | 58.15 | 51.04 | 53.27 | 66.90 |
| **50µM** | 53.04 | 49.57 | 50.62 | 51.41 | 65.69 |
| **100µM** | 42.79 | 48.25 | 46.57 | 49.62 | 62.39 |
| **^[c]^ IC50 value** | 11.16 | 15.54 | 3.26 | 5.54 | >100 |

^[a]^ The result are expressed as (-) Control=Cell and medium ^[b]^ DMSO Control =0.1% DMSO

^[c]^ IC_50_: Half maximal inhibitory concentration (μg/ml)

**Table S3.** Cytotoxic activities and IC_50_ values of compounds with %viability rates at different doses in normal colon (CCD-18Co) cells by MTT assay

| **CCD-18Co cell** | **Compound 1** | **Compound 3a** | **Compound 3b** | **Compound 3c** | **Compound 3d** |
| --- | --- | --- | --- | --- | --- |
| **^[a]^ (-) Control** | 100 | 100 | 100 | 100 | 100 |
| **^[b]^ DMSO Control** | 93.37 | 99.33 | 90.59 | 88.57 | 70.52 |
| **1.56µM** | 99.67 | 103.12 | 97.86 | 92.64 | 96.82 |
| **3.12µM** | 96.34 | 102.10 | 97.98 | 93.84 | 103.58 |
| **6.25µM** | 98.88 | 101.11 | 101.49 | 94.70 | 102.74 |
| **12.5µM** | 99.33 | 100.25 | 100.17 | 95.66 | 102.34 |
| **25µM** | 96.55 | 102.87 | 104.50 | 96.26 | 102.80 |
| **50µM** | 100.43 | 100.26 | 99.41 | 95.72 | 108.04 |
| **100µM** | 100.94 | 102.12 | 98.56 | 102.52 | 102.91 |

^[a]^ The result are expressed as (-) Control=Cell and medium ^[b]^ DMSO Control =0.1% DMSO

**Table S4.** Cytotoxic activities and IC_50_ values of compounds with %viability rates at different doses in colon cancer (DLD-1) cells by WST-8 assay

| **DLD-1 cell** | **Compound 1** | **Compound 3a** | **Compound 3b** | **Compound 3c** | **Compound 3d** |
| --- | --- | --- | --- | --- | --- |
| **^[a]^ (-) Control** | 100 | 100 | 100 | 100 | 100 |
| **^[b]^ DMSO Control** | 108.92 | 108.92 | 108.92 | 108.92 | 108.92 |
| **6.25µM** | 130.45 | 90.36 | 126.13 | 83.06 | 98.20 |
| **12.5µM** | 70.63 | 75.77 | 112.61 | 66.58 | 93.87 |
| **25µM** | 61.35 | 66.40 | 83.06 | 60.00 | 67.57 |
| **50µM** | 56.22 | 64.23 | 77.21 | 52.61 | 64.41 |
| **100µM** | 41.98 | 58.92 | 72.07 | 45.95 | 63.24 |
| **^[c]^ IC50 value** | 11.09 | >100 | >100 | 17.95 | >100 |

^[a]^ The result are expressed as (-) Control=Cell and medium ^[b]^ DMSO Control =0.1% DMSO

^[c]^ IC_50_: Half maximal inhibitory concentration (μg/ml)

**Table S5.** Cytotoxic activities and IC_50_ values of compounds with %viability rates at different doses in colon cancer (HT-29) cells by WST-8 assay

| **HT29 cell** | **Compound 1** | **Compound 3a** | **Compound 3b** | **Compound 3c** | **Compound 3d** |
| --- | --- | --- | --- | --- | --- |
| **^[a]^ (-) Control** | 100 | 100 | 100 | 100 | 100 |
| **^[b]^ DMSO Control** | 104.79 | 104.79 | 104.79 | 104.79 | 104.79 |
| **6.25µM** | 84.45 | 103.89 | 104.09 | 88.63 | 93.92 |
| **12.5µM** | 70.19 | 75.07 | 96.41 | 74.88 | 91.43 |
| **25µM** | 65.40 | 64.01 | 90.93 | 72.38 | 83.25 |
| **50µM** | 62.71 | 63.61 | 83.05 | 69.89 | 79.26 |
| **100µM** | 62.01 | 63.01 | 75.17 | 68.10 | 64.41 |
| **^[c]^ IC50 value** | >100 | >100 | >100 | >100 | >100 |

^[a]^ The result are expressed as (-) Control=Cell and medium ^[b]^ DMSO Control =0.1% DMSO

^[c]^ IC_50_: Half maximal inhibitory concentration (μg/ml)

**Table S6.** Selectivity index (SI) values of compounds in colon cancer cells (DLD-1 and HT-29) compared to normal colon cells (CCD-18Co) by MTT assay

| **Compounds** | **DLD-1 IC₅₀ (µM)** | **SI >** | **HT-29 IC₅₀ (µM)** | **SI >** |
| --- | --- | --- | --- | --- |
| **Compound 1** | 9.63 | 10.38 | 11.16 | 8.96 |
| **Compound 3a** | >100 | - | 15.54 | 6.44 |
| **Compound 3b** | 15.54 | 6.44 | 3.26 | 30.67 |
| **Compound 3c** | 3.94 | 25.38 | 5.54 | 18.05 |
| **Compound 3d** | >100 | - | >100 | - |

**Note:** A selectivity index (SI) value greater than 2 is generally considered the threshold for clinically meaningful selectivity.

**Table S7.** Primer sequences, accessions, lengths and melting temperature (^o^C) of the genes used in qRT-PCR analysis in colon cancer cells

| Genes | Primer Sequences (5’-3’) | Accessions | Lengths | Temperature (^o^C) |
| --- | --- | --- | --- | --- |
| β-Actin F | CTTCGCGGGCGACGAT | NM_001101.5 | 103 | 59 |
| β-Actin R | CACATAGGAATCCTTCTGACCCAT |  |  |  |
| BAX F | TCATGGGCTGGACATTGGAC | NM_001291428.1 | 114 | 59 |
| BAX R | GAGACAGGGACATCAGTCGC |  |  |  |
| BCL-2 F | ATCTGGGCCACAAGTGAAGTC | NM_000633.2 | 209 | 59 |
| BCL-2 R | TTCGACGTTTTGCCTGAAGAC |  |  |  |
| P53 F | GTTTTCCCCTCCCATGTGCTC | NM_001126112.2 | 170 | 60 |
| P53 R | CAGTCTGGCCAATCCAGGGAAG |  |  |  |
| CASP-3 F | GGCGCTCTGGTTTTCGTTAAT | NM_001354777.1 | 239 | 59 |
| CASP-3 R | CGACATCTGTACCAGACCGA |  |  |  |
| CASP-8 F | ATGAGCTGGGCTGAAGCAAA | NM_001228.4 | 240 | 60 |
| CASP-8 R | TCCTTCTCCCAGGATGACCC |  |  |  |
| CASP-9 F | AGGCCCCATATGATCGAGGA | NM_001229.5 | 193 | 60 |
| CASP-9 R | TCGACAACTTTGCTGCTTGC |  |  |  |
| SOD-1 F | ACAAAGATGGTGTGGCCGAT | NM_000454.5 | 162 | 59 |
| SOD-1 R | AACGACTTCCAGCGTTTCCT |  |  |  |
| SOD-2 F | GCCCTGGAACCTCACATCAA | NM_001322816.2 | 133 | 60 |
| SOD-2 R | CAGCCTGGAACCTACCCTTG |  |  |  |
| CAT F | AGTGATCGGGGGATTCCAGA | NM_001752.4 | 159 | 60 |
| CAT R | AAGTCTCGCCGCATCTTCAA |  |  |  |
| GSS F | AAGGCGAACTAGTGTTGGGA | NM_000178.4 | 176 | 59 |
| GSS R | AGAGCGTGAATGGGGCATAG |  |  |  |
