# Supplementary material for: Synthesis and Characterization of Sulfonamide‐Schiff Bases, and Investigation of Cytotoxic, Antioxidant, HDAC, and Apoptotic Activities in Human Colon Cancer Cells (DLD‐1 and HT‐29)
Source: Arch Pharm (Weinheim). 2026 Apr 10;359(4):e70235. doi: 10.1002/ardp.70235 (PMC13068298; doi:10.1002/ardp.70235)
Supplement: Supplementary file 2 — Supporting File 2 [file ARDP-359-e70235-s001.doc]

**Supplemental Material: Novel Compounds and Biological Screening Results**

**Synthesis and characterization of sulfonamide-Schiff bases, and investigation of cytotoxic, antioxidant, HDAC and apoptotic activities in human colon cancer cells (DLD-1 and HT-29)**

Seda Mesci^1a^*, Berna Kocaman^1b^, Aliye Gediz Erturk^c^*, Emine Bagdatli^c^, Burak Yazgan^d^, Tuba Yildirim^b,e^

^a^ Project Coordination and Guidance Office, Rectorate, Hitit University, 19100, Çorum, Turkey

^b^ Department of Biotechnology, Institute of Science, Amasya University, 05100 Amasya, Turkey

^c^ Department of Chemistry, Faculty of Science and Arts, Ordu University, 52200, Ordu, Turkey

^d^ Department of Medical Services and Techniques, Sabuncuoğlu Serefeddin Health Services Vocational School, Amasya University, 05100, Amasya, Turkey

^b,e^ Department of Biology, Faculty of Art and Science, Amasya University, 05100 Amasya, Turkey

| Seda Mesci | : [sedamesci@hitit.edu.tr](mailto:sedamesci@hitit.edu.tr), 0000-0002-5440-302X |
| --- | --- |
| Berna Kocaman | : [bernakocaman111@gmail.com](mailto:bernakocaman111@gmail.com), 0000-0002-5578-8697 |
| Aliye Gediz Erturk | : [aliyerturk@odu.edu.tr](mailto:aliyerturk@odu.edu.tr), 0000-0003-0831-7056 |
| Emine Bagdatli | : ebagdatli@odu.edu.tr, 0000-0001-5946-2988 |
| Burak Yazgan | : [burak.yazgan@amasya.edu.tr](mailto:burak.yazgan@amasya.edu.tr), 0000-0003-0717-7768 |
| Tuba Yildirim | : [tuba.yildirim@amasya.edu.tr](mailto:tuba.yildirim@amasya.edu.tr), 0000-0002-5578-8697 |

^1^ Both authors are co-first authors.

^a^* Corresponding author (Biology)

Phone: +90 364 2192892 e-mail: [sedamesci@hitit.edu.tr](mailto:sedamesci@hitit.edu.tr)

^c^* Corresponding author (Chemistry)

Phone: +90 262 6053011 e-mail: [aliyerturk@odu.edu.tr](mailto:aliyerturk@odu.edu.tr)

| **Compound No.** | **InChI** | **Biological Activity (IC50)** | | | |
| --- | --- | --- | --- | --- | --- |
|  |  | **MTT assay (DLD-1 cell)** | **MTT assay (HT-29 cell)** | **WST-8 assay (DLD-1 cell)** | **WST-8 assay (HT-29 cell)** |
| Compound 1 | InChI=1S/C11H12N4O3S/c1-18-11-7-6-10(13-14-11)15-19(16,17)9-4-2-8(12)3-5-9/h2-7H,12H2,1H3,(H,13,15) | 9.63 μM | 11.16 μM | 11.09 μM | 11.36 μM |
| Compound 3a | InChI=1S/C22H24N4O3S/c1-22(2,3)17-7-5-16(6-8-17)15-23-18-9-11-19(12-10-18)30(27,28)26-20-13-14-21(29-4)25-24-20/h5-15H,1-4H3,(H,24,26)/b23-15+ | 14.83 μM | 15.54 μM | 15.14 μM | 11.37 μM |
| Compound 3b | InChI=1S/C18H15N5O5S/c1-28-18-11-10-17(20-21-18)22-29(26,27)15-8-6-14(7-9-15)19-12-13-4-2-3-5-16(13)23(24)25/h2-12H,1H3,(H,20,22)/b19-12+ | 15.54 μM | 3.26 μM | 17.23 μM | 26.36 μM |
| Compound 3c | InChI=1S/C15H13N5O3S2/c1-23-14-7-6-13(18-19-14)20-25(21,22)12-4-2-11(3-5-12)17-10-15-16-8-9-24-15/h2-10H,1H3,(H,18,20)/b17-10+ | 3.94 μM | 5.54 μM | 17.95 μM | 11.11 μM |
| Compound 3d | InChI=1S/C19H15N5O3S2/c1-27-18-11-10-17(22-23-18)24-29(25,26)14-8-6-13(7-9-14)20-12-19-21-15-4-2-3-5-16(15)28-19/h2-12H,1H3,(H,22,24)/b20-12+ | 25.68 μM | 15.58 μM | 17.69 μM | 39.27 μM |
